# Integrative analysis reveals early epigenetic alterations in high-grade serous ovarian carcinomas

**DOI:** 10.1038/s12276-023-01090-1

**Published:** 2023-10-02

**Authors:** Hidenori Machino, Ai Dozen, Mariko Konaka, Masaaki Komatsu, Kohei Nakamura, Noriko Ikawa, Kanto Shozu, Ken Asada, Syuzo Kaneko, Hiroshi Yoshida, Tomoyasu Kato, Kentaro Nakayama, Vassiliki Saloura, Satoru Kyo, Ryuji Hamamoto

**Affiliations:** 1 Cancer Translational Research Team, RIKEN Center for Advanced Intelligence Project, 1-4-1 Nihonbashi, Chuo-ku, Tokyo 103-0027 Japan; 2 Division of Medical AI Research and Development, National Cancer Center Research Institute, 5-1-1 Tsukiji, Chuo-ku, Tokyo 104-0045 Japan; 3 Department of Obstetrics and Gynecology, Keio University School of Medicine, 35 Shinanomachi, Shinjyuku-ku, Tokyo 160-8582 Japan; 4 Genomics Unit, Keio Cancer Center, Keio University School of Medicine, 35 Shinanomachi, Shinjyuku-ku, Tokyo 160-8582 Japan; 5 Department of Obstetrics and Gynecology, University of Toyama, 2630 Sugitani, Toyama-shi, Toyama 930-0152 Japan; 6 Division of Diagnostic Pathology, National Cancer Center Hospital, 5-1-1 Tsukiji, Chuo-ku, Tokyo 104-0045 Japan; 7 Department of Gynecology, National Cancer Center Hospital, 5-1-1 Tsukiji, Chuo-ku, Tokyo 104-0045 Japan; 8 Department of Obstetrics and Gynecology, Shimane University Faculty of Medicine, 89-1 Enyacho, Izumo-shi, Shimane 693-8501 Japan; 9 Center for Cancer Research, National Cancer Institute, Bethesda, MD 20892 USA

**Keywords:** Ovarian cancer, Gene regulatory networks

## Abstract

High-grade serous ovarian carcinoma (HGSOC) is the most lethal gynecological malignancy. To date, the profiles of gene mutations and copy number alterations in HGSOC have been well characterized. However, the patterns of epigenetic alterations and transcription factor dysregulation in HGSOC have not yet been fully elucidated. In this study, we performed integrative omics analyses of a series of stepwise HGSOC model cells originating from human fallopian tube secretory epithelial cells (HFTSECs) to investigate early epigenetic alterations in HGSOC tumorigenesis. Assay for transposase-accessible chromatin using sequencing (ATAC-seq), chromatin immunoprecipitation sequencing (ChIP-seq), and RNA sequencing (RNA-seq) methods were used to analyze HGSOC samples. Additionally, protein expression changes in target genes were confirmed using normal HFTSECs, serous tubal intraepithelial carcinomas (STICs), and HGSOC tissues. Transcription factor motif analysis revealed that the DNA-binding activity of the AP-1 complex and GATA family proteins was dysregulated during early tumorigenesis. The protein expression levels of JUN and FOSL2 were increased, and those of GATA6 and DAB2 were decreased in STIC lesions, which were associated with epithelial-mesenchymal transition (EMT) and proteasome downregulation. The genomic region around the FRA16D site, containing a cadherin cluster region, was epigenetically suppressed by oncogenic signaling. Proteasome inhibition caused the upregulation of chemokine genes, which may facilitate immune evasion during HGSOC tumorigenesis. Importantly, MEK inhibitor treatment reversed these oncogenic alterations, indicating its clinical effectiveness in a subgroup of patients with HGSOC. This result suggests that MEK inhibitor therapy may be an effective treatment option for chemotherapy-resistant HGSOC.

## Ovarian cancer: The significance of epigenetic changes

Analyzing epigenetic effects on gene activity, which are due to changes other than mutations in the primary DNA sequence of genes, reveals new insights into the most lethal form of ovarian cancers. Epigenetic effects are due to chemical modifications of DNA, RNA and associated proteins that can influence the activity of genes and the proteins they code for. Researchers in Japan led by Hidenori Machino and Ryuji Hamamoto at the RIKEN Center for Advanced Intelligence Project, Tokyo, analyzed epigenetic alterations in a series of human cells modelling stages of high-grade serous ovarian carcinoma (HGSOC). They identified significant changes in the activity of several specific genes, RNAs and proteins. The insights prompted the researchers to undertake cell-based studies that suggested drugs inhibiting key cell regulatory enzymes known as MEK proteins could be used as new treatments.

## Introduction

In the past decade, comprehensive genome analyses, such as whole exome and whole genome sequencing, have successfully identified driver mutations in several subgroups of malignancies^1^. However, many cases remain untreatable due to the lack of targetable mutations, posing a challenge in precision oncology medicine^2^. Recently, comprehensive epigenomic analysis has been extensively used in biology, and its clinical application in cancer therapy has been actively explored^3,4^. An epigenomic analysis is expected to overcome the limitations of conventional genome analysis and offer additional opportunities to identify novel target genes.

Unlike genomic sequences, which are identical across different cell types from the same donor, epigenomic profiles differ substantially depending on cell type^5^. Given that cancer cells arise from their normal cell-of-origin, purified cell-of-origin samples of each cancer type are required to perform precise epigenomic analysis. Although sample collection is challenging, purified cell-of-origin samples can be ideal normal controls to elucidate cancer-type-specific tumorigenic mechanisms^6,7^.

High-grade serous ovarian carcinoma (HGSOC) is the most aggressive histological type of ovarian cancer^8^. The majority of HGSOCs originate from human fallopian tube secretory epithelial cells (HFTSECs), as evidenced by the presence of serous tubal intraepithelial carcinomas (STICs)^9,10^. Because our research group has established techniques for harvesting primary cultured HFTSECs and generating immortalized cells and tumorigenic cells from HFTSECs, we selected HGSOC as our research target for integrative epigenomic analysis^11^.

Approximately half of HGSOC cases are phenotypically classified as homologous recombination (HR)-deficient, where the DNA repair pathway for double-strand breaks is impaired, resulting in high sensitivity to a PARP inhibitor^12,13^. Cell-of-origin research on HR-deficient HGSOCs has been frequently reported. For example, tumorigenic mouse models for HR-deficient HGSOCs have already been established (e.g., *Pax8-rtTA*, *TetO-Cre*, *Brca1*^*loxP/loxP*^, *Trp53mut*, *Pten*^*loxP/loxP*^, *Ovgp1-iCreER*^*T2*^, *Brca1*^*loxP/loxP*^, *Trp53*^*loxP/loxP*^, *Rb1*^*loxP/loxP*^, and *Nf1* ^*loxP/loxP*^)^14,15^.

In contrast, the remaining HGSOCs are HR-proficient types. To date, there have been no established mouse or human tissue-derived cell models of HR-proficient HGSOC. In addition, HR-proficient HGSOCs often exhibit chemoresistance and poor prognosis, leaving many patients with unmet medical needs^16^. Moreover, recent comprehensive copy number analysis of HGSOCs revealed that copy number signatures with aberrant Ras signaling were linked to the worst prognosis^17^. Thus, we performed an integrative epigenomic analysis of HR-proficient HGSOCs using human tissue-derived HGSOC model cell samples with Ras activation.

We utilized a series of stepwise HGSOC model cells previously established by Nakamura et al., in which HFTSECs were genetically engineered to acquire tumorigenic potential^11^. These samples fulfilled the following criteria for precise epigenomic analysis: (i) derived from purified human cell-of-origin HGSOC, (ii) possessing an HR-proficient HGSOC genetic background, and (iii) having tumorigenic potential for HGSOC.

In this study, we performed multiomics analysis using RNA sequencing (RNA-seq), assay for transposase-accessible chromatin using sequencing (ATAC-seq), and chromatin immunoprecipitation sequencing (ChIP-seq) for H3K27Ac to identify early epigenetic alterations in HGSOC tumorigenesis using a series of stepwise HGSOC model cells.

## Materials and methods

### Patients and clinical specimens

This study was approved by the Ethics Committee of the National Cancer Center, Tokyo, Japan (approval ID: 2016-496) and Shimane University, Shimane, Japan (approval ID: 20070305-1 and 2007305-2). Written informed consent was obtained from patients who participated in this study in accordance with the Declaration of Helsinki.

For long-term storage, fresh tissues were frozen in liquid nitrogen immediately after sampling and stored at −80 °C. The tissue samples were embedded into an optimal cutting temperature (OCT) compound, followed by frozen sectioning.

### Isolation of HFTSECs

HFTSECs were isolated as previously described^11^. Briefly, fresh fimbriae were washed with phosphate-buffered saline (PBS), resected longitudinally, plated on 25 cm^2^ dishes containing Dulbecco’s modified Eagle’s medium (DMEM) with 5% fetal bovine serum (FBS), and rocked gently at 25 °C for 48 h to dissociate the epithelial cells. The fimbriae and dissociated cells were cultured in a 37 °C incubator with 5% CO_2_. After 7 d, the fimbriae were removed, and the adherent epithelial cells were continuously cultured until they reached 60–70% confluency. These primary cultured HFTSECs were genetically engineered for immortalization or harvested for RNA-seq and ATAC-seq sampling.

### Development of immortalized HFTSEC and HGSOC model cells

Immortalized HFTSEC and HGSOC model cells were provided by Dr. Kyo et al. Detailed development methods have been previously described^11^. Briefly, primary HFTSECs were immortalized by adding *hTERT*, *CCND1*, and *CDK4*^R24C^ without antibiotic selection. Subsequently, stepwise gene editing using lentiviral vectors and antibiotics was performed in immortalized HFTSECs: dominant-negative *TP53* with 250 μg/ml G418, *KRAS*^V12^ with 50 μg/ml hygromycin-B, Myr-*AKT1* with 0.5 μg/ml puromycin and *MYC* with 0.5 μg/ml puromycin.

### Cell lines and culture conditions

CaOV3 and ES2 cells were purchased from the American Type Culture Collection (ATCC, Manassas, VA, USA). JHOS2, JHOS4, and OVCAR3 cells were purchased from the Riken Cell Bank (Ibaraki, Japan). OVSAHO and TYK-nu cells were purchased from the Japanese Collection of Research Bioresources Cell Bank (JCRB, Osaka, Japan). SNU8 cells were purchased from the Korean Cell Line Bank (KCLB, Seoul, South Korea). CaOV3 and SNU8 cells were cultured in DMEM with 10% FBS. JHOS2 and JHOS4 cells were cultured in DMEM/Ham F12 medium with 10% FBS and 0.1 mM nonessential amino acids (NEAA). ES2 and OVSAHO cells were cultured in RPMI 1640 medium with 10% FBS, and OVCAR3 cells were maintained in RPMI 1640 medium with 20% FBS and 0.1% insulin. TYK-nu cells were cultured in Eagle’s minimum essential medium (EMEM) with 10% FBS. All the cell lines were authenticated using short tandem repeat (STR) profiling (Supplementary Table 1). We routinely looked for *Mycoplasma* contamination in these cell lines using an e-Myco Mycoplasma PCR detection kit (25235; iNtRON Biotechnology, Korea).

### Reverse-transcription quantitative real-time PCR (qRT‒PCR)

Total RNA from the cell lines was extracted using QIAzol lysis reagent and an RNeasy Plus mini kit (73404; Qiagen, Hilden, Germany), and cDNA was synthesized using the PrimeScript RT reagent kit (RR037A; TaKaRa Bio, Kusatsu, Japan) according to the manufacturer’s instructions. qRT‒PCR was performed using TB Green Premix Ex Taq II (RR820A; TaKaRa Bio) and the CFX96 touch system (Bio-Rad, Hercules, CA, USA). mRNA expression levels were normalized to those of *GAPDH* mRNA, the internal control, using the ΔCq method. Detailed information regarding the primers used in this study is provided in Supplementary Table 2.

### RNA-seq analysis

We performed total RNA extraction, DNase I treatment, mRNA isolation using magnetic beads with oligo (dT), and mRNA fragmentation. Next, cDNA was synthesized using mRNA fragments as templates^18^. The cDNA was connected to adapter sequences and processed for PCR amplification. The Agilent 2100 Bioanalyzer and ABI StepOnePlus Real-Time PCR system were used for the quantification and qualification of the sample library, respectively. The library was then sequenced on an Illumina BGISEQ-500 or Illumina NovaSeq6000. In the data filtering step, adaptor sequences, contaminations, and low-quality reads were eliminated from the raw reads.

RNA-seq reads were aligned with the human reference genome NCBI build hg38 using STAR^19^. Tags per million clean tags (TPM) were calculated using RNA-seq by expectation-maximization (RSEM)^20^, and differentially expressed genes (DEGs) were extracted using edgeR^21^. A false discovery rate (FDR) of less than 0.05 was considered statistically significant. Gene Ontology (GO) analysis was performed on DAVID (the Database for Annotation, Visualization, and Integrated Discovery; v.6.8) (https://david.ncifcrf.gov/summary.jsp).

### ATAC-seq analysis

ATAC-seq was performed as previously described by Active Motif (Carlsbad, CA, USA)^18^. FASTQ files were processed for adapter sequence trimming and mapped to NCBI build hg19 using bowtie2 (v.2.3.4.2) with the option of very-sensitive × 2000 and PCR duplicate removal. Mapping quality was assessed using DRaw and Observe Multiple enrichment Profiles and Annotation (DROMPA)^22^. Peaks were called using MACS2 (v.2.1.2) with the option -f BAM -g hs -q 0.01 --nomodel --shift -75 --extsize 150 -B and further filtered with *p-value* < 10^−10^ ^23^. The peak raw counts were normalized using quantile normalization. Transcription factor (TF) motif enrichment analysis was performed as previously described^24^. Briefly, a peak versus motif matrix was generated using HOMER (v.4.11), which combined ATAC-seq peaks and JASPAR core nonredundant position frequency matrices for vertebrates. A peak versus motif matrix and a peak versus intensity matrix were integrated into the TF motif enrichment matrix scores using the Module Map algorithm of Genomica (v.1.0). The results of the TF motif enrichment analysis are summarized in Supplementary Table 3.

### ChIP-seq analysis

Cell lines were fixed with 1% formaldehyde and collected in ice-cold PBS containing 1 mM phenylmethylsulfonyl fluoride (PMSF). Nuclei were prepared, and chromatin was digested according to the manufacturer’s instructions (#9003; Cell Signaling Technology, Danvers, MA, USA). Nuclei pellets were resuspended in ChIP buffer (50 mM Tris–HCl [pH 8.0], 150 mM NaCl, 1% Triton X-100, 0.5% IGEPAL CA-630, 5 mM EDTA [pH 8.0], 1 mM PMSF, and protease inhibitor cocktail). Samples were sonicated using a Bioruptor II (BR2006A; BM Equipment, Tokyo, Japan) to generate DNA fragments of ~200 bp. Antibodies against H3K27ac (#4729, Lot GR3252404; Abcam, Cambridge, UK) were added to the DNA fragments and incubated in an ultrasonic water bath at 4 °C for 30 min. After centrifugation, the supernatant was incubated with FG Beads HM Protein G (TAB8848N3173; Tamagawa Seiki, Nagano, Japan) at 4 °C for 30 min. Beads were washed twice with ChIP buffer, wash buffer (50 mM Tris–HCl [pH 8.0], 300 mM NaCl, 1% Triton X-100, 0.1% SDS, 0.1% Na-deoxycholate, and 5 mM EDTA [pH 8.0]), and LiCl buffer (50 mM Tris–HCl [pH 8.0], 250 mM LiCl, 1% Triton X-100, 0.5% Na-deoxycholate, and 5 mM EDTA [pH 8.0]). Immunoprecipitated chromatin was eluted and reverse-crosslinked according to the manufacturer’s instructions (#9003; Cell Signaling Technology). Immunoprecipitated DNA was purified using the QIAquick PCR purification kit (#28106; Qiagen). DNA libraries were generated using a QIAseq ultralow input library kit (#180492; Qiagen). The size of the DNA libraries was determined and quantified using qPCR (E7630; New England Biolabs, Ipswich, MA, USA) using an Agilent 2100 Bioanalyzer. The DNA libraries were sequenced using an Illumina HiSeq 4000 sequencer.

### Bioinformatic analysis

Two independent datasets (GSE18521 and GSE26712), which included the microarray data of human ovarian surface epithelial (HOSE) and HGSOC samples, were obtained from the Gene Expression Omnibus (GEO). Differential expression analysis between HOSE and HGSOC was performed using the GEO2R pipeline, in which GEOquery and limma were used with default parameters. The *MAF* mRNA expression levels were fitted to a log2 scale.

Gene expression, copy number, and clinical data of patients with HGSOC from The Cancer Genome Atlas (TCGA) project were sourced from the cBioPortal for cancer genomics. Survival curves were visualized using the Kaplan–Meier method and analyzed using the log-rank test. For each gene, receiver operating characteristic (ROC) curves were utilized to determine optimal cutoff values that effectively segregated the expression data into low- and high-expression groups.

### Immunohistochemistry

For formalin-fixed paraffin-embedded tissue specimens, tissue sections were deparaffinized, and antigen retrieval was performed at 110 °C for 5 min in Target Retrieval Solution x10 (S1699; Agilent Dako). Tissues were incubated with 3% H_2_O_2_ (086-07445; Fujifilm Wako Pure Chemical Corporation, Tokyo, Japan) diluted in methanol for 10 min to reduce endogenous peroxidase activity, followed by blocking with Blocking One Histo (06349-64; Nacalai Tesque, Kyoto, Japan) for 10 min. Fixed tissue sections were incubated with primary antibodies at 4 °C overnight, followed by incubation with EnVision+ System-HRP-Labeled polymer anti-rabbit secondary antibodies (K4003; Agilent Dako) at 25 °C for 30 min. For detection of horseradish peroxidase (HRP) reactions, the EnVision DAB+ Substrate Chromogen System (K3467; Agilent Dako) was used. Finally, tissue specimens were stained with Mayer’s hematoxylin solution (30011; Muto Pure Chemicals, Tokyo, Japan) for 10 s.

The following primary antibodies were used: anti-DAB2 rabbit antibody (sc-136964; Santa Cruz Biotechnology, Dallas, TX, USA; dilution 1:200), anti-FOSL2 rabbit antibody (#19967; Cell Signaling Technology; dilution 1:7500), anti-GATA6 rabbit antibody (#5851; Cell Signaling Technology; dilution 1:400), anti-JUN rabbit antibody (#9165; Cell Signaling Technology; dilution 1:300), anti-phospho-JUN rabbit antibody (#3270; Cell Signaling Technology; dilution 1:200), anti-p53 rabbit antibody (#2527; Cell Signaling Technology; dilution 1:160), and anti-PAX8 rabbit antibody (#10336-1-AP; Proteintech, Rosemont, IL, USA; dilution 1:1000).

### siRNA transfection

siRNA transfection was performed using Lipofectamine RNAiMax transfection reagent (13778-150; Thermo Fisher Scientific) following the manufacturer’s instructions. AccuTarget negative control siRNA (SN-1013; Bioneer, Oakland, CA, USA), DANCR siRNA#1 (n272698; Thermo Fisher Scientific), DANCR siRNA#2 (custom siRNA; sense: GUCUCUUACGUCUGCGGAAdTdT, antisense: UUCCGCAGACGUAAGAGACdTdT, Sigma-Aldrich, St. Louis, MO, USA), MAF siRNA (SASI_Hs01_00202727; Sigma-Aldrich), GATA6 siRNA (SASI_Hs02_00339287; Sigma-Aldrich), and DAB2 siRNA (SASI_Hs01_00161136; Sigma-Aldrich) were used.

### Colony formation assays

Cells were plated in 6-well plates at the following concentrations: 7500 cells/well for CaOV3, 10000 cells/well for OVCAR3, 10,000 cells/well for HF1/TP53/KRAS/AKT, and 15,000 cells/well for HF1/TP53/KRAS/MYC. The culture medium was replaced every three days. The incubation periods for the different cell lines were as follows: CaOV3, 25 days; OVCAR3, 30 days; HF1/TP53/KRAS/AKT, 27 days; and HF1/TP53/KRAS/MYC, 8 days. Cells were fixed with 1% formaldehyde (252549; Sigma-Aldrich) and 1% methanol (137-01823; Fujifilm Wako Pure Chemical Corporation), stained with 0.05% crystal violet (V5265; Sigma-Aldrich) for 20 min, and then washed three times. Colony number and area were quantified using ImageJ software.

### Cell viability assays

Cells were plated in 96-well plates at a concentration of 3000 cells/well for HF1/TP53/KRAS/MYC cells and 5000 cells/well for HF1, JHOS2, JHOS4, OVCAR3, and OVSAHO cells. For trametinib treatment, all cells were plated at 1000 cells/well. Three days after treatment, 10 μl of Cell Counting Kit-8 (343-07623, Dojindo, Kumamoto, Japan) reagent was added to each well. After 2 h of reaction, cell viability was determined by measuring the absorbance at 450 nm using Multiskan FC (Thermo Fisher Scientific).

### Inhibitors

The following compounds were used under the indicated conditions: trichostatin A (TSA; S1045; Selleck Chemicals, Houston, TX, USA), carfilzomib (AG-CR1-3669; AdipoGen, San Diego, CA, USA), and trametinib (CS-0060; ChemScene, Monmouth Junction, NJ, USA).

### Quantification and statistical analysis

Each experiment was repeated at least three times and was successfully reproduced throughout the manuscript. Values are presented as the mean ± standard deviation (SD). Unpaired Student’s *t* test with a two-tailed distribution was used for comparisons between two groups. One-way analysis of variance (ANOVA) was used to compare more than two groups. Pearson’s correlation coefficient was used for the correlation analysis. A *p*-value of less than 0.05 was considered statistically significant unless otherwise specified.

## Results

### Transcriptome analysis captures early tumorigenic changes in HGSOC

**Fig. 1:**
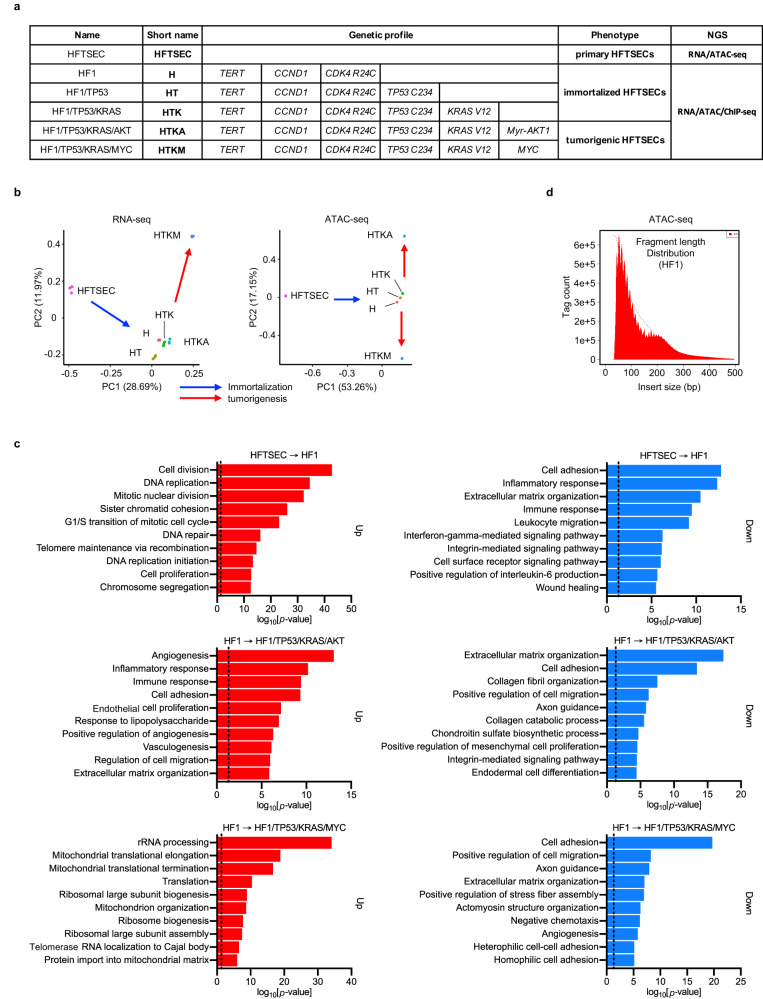
Integrative analysis of stepwise HGSOC model cells. **a** Sample list of stepwise HGSOC model cells. The genetic profile provides detailed information on gene editing. HF1/TP53/KRAS/AKT and HF1/TP53/KRAS/MYC cells exhibit tumorigenic capacities in mouse xenograft experiments. RNA-seq (technical replicates; *n* = 3) and ATAC-seq (no replication) data are available for all samples. ChIP-seq (no replication) data are available in immortalized HFTSEC and tumorigenic HGTSEC samples. **b** Principal component analysis (PCA) indicates stepwise changes in HGSOC model cells in both RNA-seq and ATAC-seq. Tags per million tags (TPM) of each transcript (RNA-seq) and peak (ATAC-seq) are processed in PCA. **c** Gene Ontology (GO) analysis of upregulated differentially expressed genes (DEGs) (left, *n* = 1000) and downregulated DEGs (right, *n* = 1000) obtained from RNA-seq data. Dotted lines indicate the position of *p* = 0.05. upper: HFTSEC → HF, middle: HF1 → HF1/TP53/KRAS/AKT, lower: HF1 → HF1/TP53/KRAS/MYC. **d** Fragment length analysis of ATAC-seq data in the HF1 sample. A peak approximately 200 bp corresponds to where Tn5 inserted around a single nucleosome.

Integrative analysis was performed using human-derived HGSOC model cell samples (Fig. 1a), including HFTSEC, HF1 (H), HF1/TP53 (HT), HF1/TP53/KRAS (HTK), HF1/TP53/KRAS/AKT (HTKA), and HF1/TP53/KRAS/MYC (HTKM), wherein stepwise genetic transduction was conducted. The HF1 sample was an immortalized cell line established by overexpressing *TERT*, *CCND1*, and *CDK4 R24C* in primary cultured human fallopian tube secretory cells. The HF1/TP53 sample expressed dominant-negative *TP53 C234* in HF1, recapitulating STIC, which is characterized by aberrant overexpression of mutant p53 protein expression.

As we previously reported, an immortalized human fallopian tube secretory cell line gained tumorigenic potential by dominant-negative p53 induction, Ras activation, and Myr-AKT1 or MYC overexpression (Supplementary Fig. 1a, b)^11^. Therefore, we prepared the following stepwise tumorigenic model cell samples: HF1/TP53/KRAS samples expressing KRAS V12, HF1/TP53/KRAS/AKT samples with KRAS V12 and Myr-AKT1 overexpression, and HF1/TP53/KRAS/MYC samples with KRAS V12 and MYC overexpression. Among these, the HF1/TP53/KRAS/AKT and HF1/TP53/KRAS/MYC samples successfully induced HGSOC tumor formation in mouse xenograft experiments.

For integrative analysis, we performed RNA-seq and ATAC-seq using HGSOC model cell samples (Fig. 1a, Supplementary Fig. 1c, d). Principal component analysis (PCA) of RNA-seq data revealed three distinct clusters in HGSOC model cells. The first cluster only comprised the HFTSEC samples. The second cluster included HF1, HF1/TP53, HF1/TP53/KRAS, and HF1/TP53/KRAS/AKT, suggesting that the immortalization step (HFTSEC to HF1) significantly influences cell identity, emphasizing the importance of evaluating the status of primary cultured cells as a normal control. HF1/TP53/KRAS/MYC samples, another tumorigenic cell, formed a distinct third cluster, suggesting that the MYC protein plays an important role in HGSOC tumorigenesis. Overall, the PCA results of RNA-seq validated the stepwise changes in HGSOC model cells from primary culture to tumorigenesis (Fig. 1b, left).

Next, we performed GO analysis using RNA-seq data. As the immortalization step (HFTSEC to HF1) and the tumorigenic step (HF1 to HF1/TP53/KRAS/MYC) showed the most significant changes in the transcriptome, our GO analysis was focused on the following three representative steps: (1) HFTSEC to HF1, (2) HF1 to HF1/TP53/KRAS/AKT, and (3) HF1 to HF1/TP53/KRAS/MYC (Fig. 1c).

The most significantly affected GO terms in upregulated DEGs were “Cell division” in the HFTSEC to HF1 step, “Angiogenesis” in the HF1 to HF1/TP53/KRAS/AKT step, and “rRNA processing” in the HF1 to HF1/TP53/KRAS/MYC step (Fig. 1c, red bars). As the HFTSEC to HF1 step was driven by typical cell cycle regulators, such as CCND1 and CDK4, it is reasonable that the GO term “Cell division” was enriched in this step. Under the activated GO term of “Angiogenesis” in HF1/TP53/KRAS/AKT samples, they showed significant upregulation of *VEGFA*, which is an important therapeutic target for the molecular-targeted drug bevacizumab. MYC protein reportedly augments global protein synthesis by stimulating ribosome biogenesis through the upregulation of ribosomal RNA and ribosomal proteins, which was also concordant with our GO analysis results (Supplementary Fig. 1e).

In contrast, downregulated DEGs in each step were commonly enriched in GO terms related to cell adhesion molecules such as “Cell adhesion” and “Extracellular matrix organization”. The majority of HGSOCs originate from fallopian tube secretory epithelial cells, which transform into STICs and subsequently disseminate to the ovarian surface. Thus, the initial tumorigenic process in HGSOCs may be characterized by the loss of epithelial cell identity due to the dysfunction of cell adhesion molecules. This hypothesis suggests that epithelial-mesenchymal transition (EMT), a hallmark of cancer, is actively involved in enabling human fallopian tube secretory cells to acquire tumorigenic potential (Fig. 1c, blue bars)^25^.

Overall, these findings are consistent with the accumulated evidence explaining early tumorigenic changes, indicating that our RNA-seq data reflected biologically relevant information on HGSOC tumorigenesis.

### TF motif analysis predicted dysregulated DNA-binding activities of AP-1 and GATA family proteins

A cell-of-origin epigenome profile serves as the optimal normal control to elucidate the mechanism by which cancer cells acquire an oncogenic phenotype because epigenetic reprogramming determines organ-specific cell fates. Therefore, we tracked epigenetic changes from a cell-of-origin signature to a malignant tumor signature by performing ATAC-seq, a comprehensive method to characterize genome-wide chromatin accessibility, using a series of HGSOC model samples from primary cultured cells to tumorigenic cells^26^.

First, we analyzed the fragment length and confirmed a nucleosome-wide peak, suggesting that ATAC-seq was successfully conducted (Fig. 1d). Importantly, PCA of ATAC-seq showed stepwise changes from primary cultured cells to tumorigenic cells, similar to RNA-seq. Furthermore, although PCA of RNA-seq failed to capture considerable differences between HF1/TP53/KRAS/AKT samples and immortalized cell samples, ATAC-seq distinguished the HF1/TP53/KRAS/AKT sample from immortalized cell samples (Fig. 1b, right). This result is reasonable because ATAC-seq has been reported to classify cancer types more accurately than RNA-seq. This finding may be attributed to the fact that distal regulatory elements demonstrate a higher level of specificity in their association with different cancer types^3^.

**Fig. 2:**
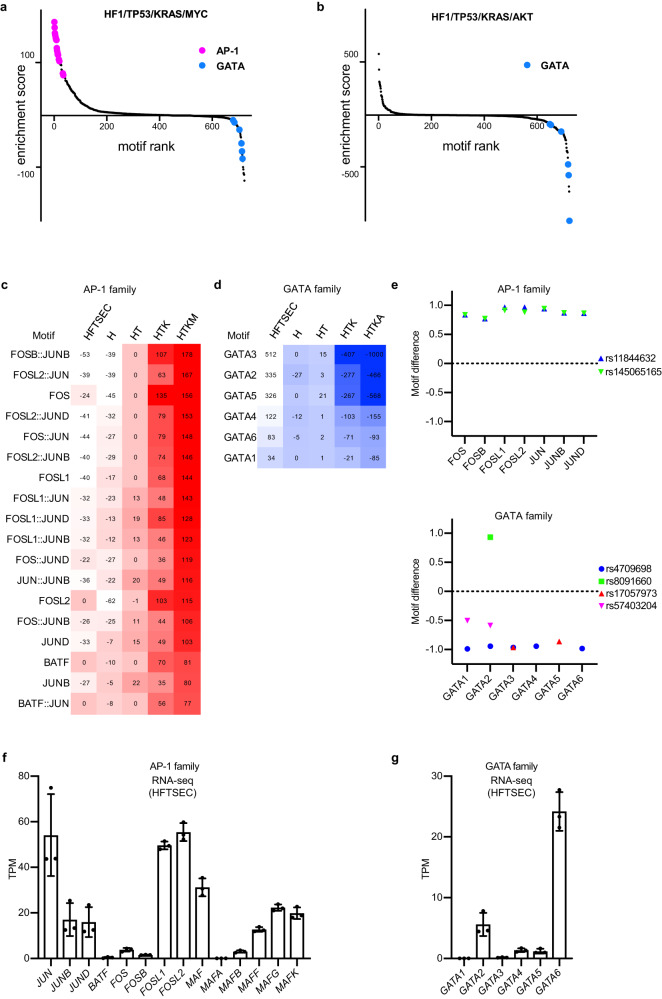
Transcription factor motif analysis predicts activation of AP-1 family proteins and suppression of GATA family proteins. **a**, **b** Rank order plots of transcription factor (TF) motif analysis using ATAC-seq data of HF1/TP53/KRAS/MYC cells and HF1/TP53/KRAS/AKT cells. These plots display the predicted DNA-binding activities of a total of 722 TF motifs. In HF1/TP53/KRAS/MYC cells, AP-1 family TFs show high activation, while GATA family TFs are suppressed in both cell lines. Jaspar core nonredundant motifs were utilized in this TF motif analysis. **c**, **d** TF motif analysis of AP-1 family genes and GATA family genes using ATAC-seq data. The heatmap shows the predicted DNA-binding activities of differentially regulated motifs. Jaspar core nonredundant motifs were utilized in this TF motif analysis. **e** TF motif analysis of AP-1 family genes and GATA family genes using GWAS data of ovarian cancer risk loci. HOCOMOCO motifs were utilized in this TF motif analysis. **f**, **g** mRNA expression levels (RNA-seq, *n* = 3) of AP-1 family genes and GATA family genes in primary human fallopian tube secretory epithelial cell (HFTSEC) samples (*n* = 3). Error bars represent the mean ± standard deviation (SD).

Next, we extracted a list of differentially accessible TFs by computing the occurrence of TF motif sequences (Jasper2018_CORE TF) within open chromatin regions^27^. Rank order plots of TF motif analysis revealed characteristic TF families whose DNA-binding activities were predicted to increase or decrease during oncogenic transformation (Fig. 2a, b). The DNA-binding scores of AP-1 family proteins, such as JUN and FOS family proteins, were continuously elevated during oncogenic transformation from primary cultured cells to HF1/TP53/KRAS/MYC cells (Fig. 2c). In contrast, the DNA binding scores of GATA family proteins showed a gradual decrease, particularly from primary cultured cells to HF1/TP53/KRAS/AKT cells (Fig. 2d). These results suggest oncogenic roles of the JUN and FOS family proteins and tumor-suppressive roles of the GATA family proteins in HGSOC tumorigenesis.

We further validated our TF motif analysis results using different data modalities. We obtained GWAS single nucleotide polymorphism (SNP) data of ovarian carcinoma risk loci and extracted SNPs that affected the TF motif sequences of AP-1 and GATA families^28^. The TF motifs of AP-1 family proteins were generated (rs11844632 and rs145065165), and those of GATA family proteins were often disturbed (rs4709698, rs17057973, and rs57403204) by ovarian carcinoma risk loci (Fig. 2e). These results support our hypothesis that AP-1 family genes are oncogenic and GATA family genes are tumor-suppressive in the early stages of HGSOC tumorigenesis.

The AP-1 complex is a heterodimer comprising AP-1 family proteins, including JUN, FOS, ATF, and MAF family proteins^29^. The GATA family comprises six proteins, GATA1–GATA6. In primary HFTSEC samples, the gene expression levels of *JUN*, *FOSL1*, *FOSL2*, and *MAF* were relatively high among AP-1 family genes (Fig. 2f), and that of *GATA6* was the highest among GATA family genes (Fig. 2g). In the TCGA HGSOC dataset, cases with copy number gain or amplification were frequently observed for the JUN family genes *FOSL1* and *FOSL2*, whereas copy number loss was dominant in *MAF* (Supplementary Fig. 2a). The copy number profile of GATA family genes is inconsistent among family members. However, *GATA6*, a highly expressed gene in HFTSEC samples, often suffers from a loss of heterozygosity (LOH) in HGSOC samples (Supplementary Fig. 2b). The following sections include the functional analysis of these genes.

### AP-1 and GATA family genes are epigenetically dysregulated during HGSOC tumorigenesis

To further validate our ATAC-seq analysis, we compared the protein expression levels between normal and cancerous cells. STIC specimens were collected from human fallopian tubes, which captured early changes in HGSOC tumorigenesis.

**Fig. 3:**
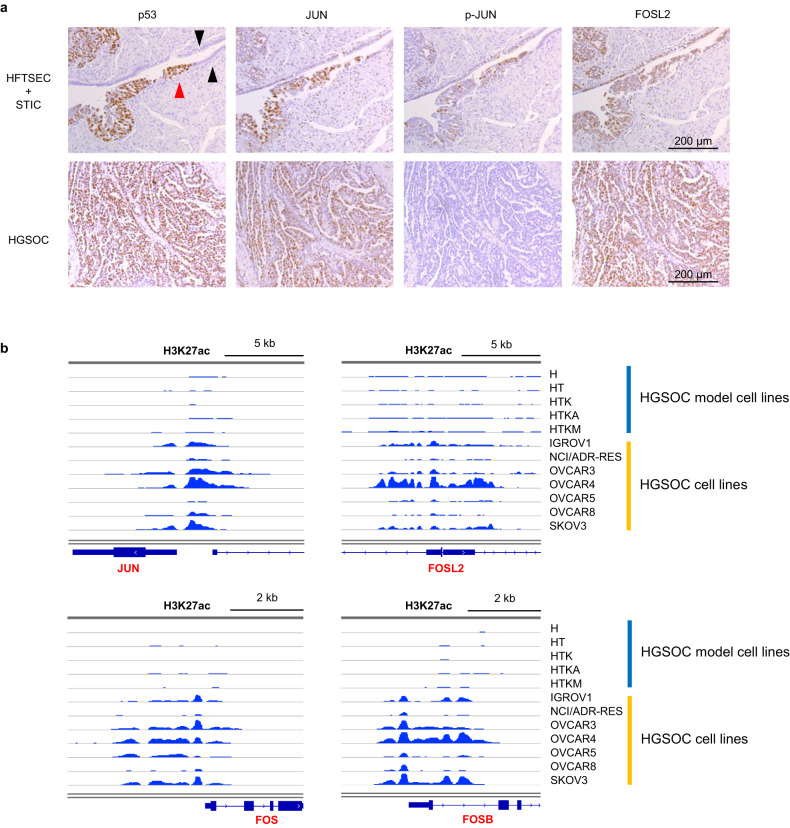
AP-1 family genes are upregulated in STIC and HGSOC. **a** Immunohistochemistry shows that JUN and FOSL2 are upregulated in STIC (upper images) and HGSOC (lower images). Phospho-JUN is augmented in only STIC, suggesting that activation of the AP-1 complex is essential in the early stage of tumorigenesis. p53 staining was used as a positive control for STIC and HGSOC. The black arrows indicate normal fallopian tube epithelial cells, while the red arrows indicate STIC lesions. Scale bars, 200 μm. **b** ChIP-seq for H3K27ac. Enhancer histone marks around the JUN, FOSL2, FOS and FOSB loci are increased in ovarian cancer cell lines.

Immunohistochemistry revealed that AP-1 family proteins, such as JUN and FOSL2, were upregulated in STIC and HGSOC lesions compared to normal fallopian tube epithelial cells. Importantly, phosphorylated JUN, which is transcriptionally active, was upregulated only in STIC lesions, suggesting that the activation of the AP-1 complex plays an essential role during the early process of HGSOC tumorigenesis (Fig. 3a).

The increase in enhancer histone marks around the JUN and FOS family gene loci in ovarian cancer cells was confirmed. ChIP-seq with antibodies against H3K27ac modification is a standard method for evaluating genome-wide enhancer profiles. Thus, we performed ChIP-seq of H3K27ac modifications in our stepwise HGSOC model cells. Additionally, we obtained publicly available ChIP-seq data of H3K27ac modifications in ovarian cancer cell lines (IGROV1, NCI/ADR-RES, OVCAR3, OVCAR4, OVCAR5, OVCAR8, and SKOV3). The enhancer histone marks were augmented in most ovarian cancer cell lines compared to stepwise HGSOC model cells, suggesting that AP-1 family genes are epigenetically upregulated in HGSOCs (Fig. 3b).

Next, we investigated the epigenetic regulation of GATA family genes, a candidate for differentially regulated TFs extracted by our ATAC-seq analysis. As shown in Fig. 2d, g, the DNA-binding affinities of GATA family members decreased during HGSOC tumorigenesis, and the mRNA expression level of *GATA6* in cell-of-origin samples was the highest among GATA family genes. Therefore, we hypothesized that *GATA6* is a potential tumor suppressor gene epigenetically downregulated in HGSOCs. In addition, GATA6 promotes the mRNA expression of *DAB2*, a known tumor suppressor gene of ovarian carcinoma^30,31^. Hence, we focused on the GATA6-DAB2 axis as a potential tumor suppressor cascade in HGSOCs.

**Fig. 4:**
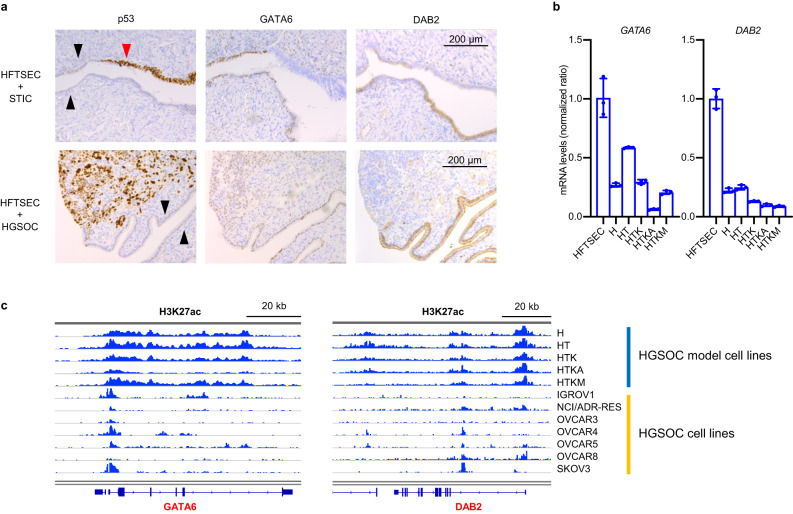
GATA family genes are downregulated in STIC and HGSOC. **a** Immunohistochemistry shows that GATA6 and DAB2 are downregulated in STIC (upper images) and HGSOC (lower images). p53 staining was used as a positive control for STIC and HGSOC. The black arrows indicate normal fallopian tube epithelial cells, while the red arrows indicate STIC lesions. Scale bars, 200 μm. **b** mRNA expression levels (RT‒qPCR, *n* = 3) of *GATA6* and *DAB2* are decreased in HGSOC model cells. Error bars represent the mean ± standard deviation (SD). **c** ChIP-seq for H3K27ac. Enhancer histone marks around the JUN, FOSL2, FOS and FOSB loci are increased in ovarian cancer cell lines.

We confirmed the downregulation of GATA6 and DAB2 expression during HGSOC tumorigenesis by comparing the protein expression levels of HFTSECs, STICs, and HGSOCs (Fig. 4a). Furthermore, in our stepwise HGSOC model cells, the mRNA expression levels of *GATA6* and *DAB2* gradually decreased during oncogenic transformation (Fig. 4b, Supplementary Fig. 2c). Likewise, H3K27ac modification around the *GATA6* and *DAB2* loci was reduced in ovarian cancer cell lines (Fig. 4c).

In summary, our integrative analysis revealed that AP-1 and GATA family genes are epigenetically dysregulated during the early stages of HGSOC tumorigenesis. The cellular effects of AP-1 and GATA family dysregulation are reported in the following sections.

### Genomic region around common chromosomal fragile site in 16D (FRA16D), containing the CDH family cluster and *MAF* gene, is epigenetically silenced in HGSOCs

As previously mentioned, GO analysis of RNA-seq data indicated that downregulated DEGs upon oncogenic transformation were enriched in the GO terms “Cell adhesion” and “Extracellular matrix organization.” This result implies that EMT is an initial step in HGSOC tumorigenesis^25^. Importantly, the AP-1 complex promotes and GATA6 inhibits EMT, suggesting that AP-1 activation and GATA6 suppression in HGSOCs trigger EMT^32,33^.

**Fig. 5:**
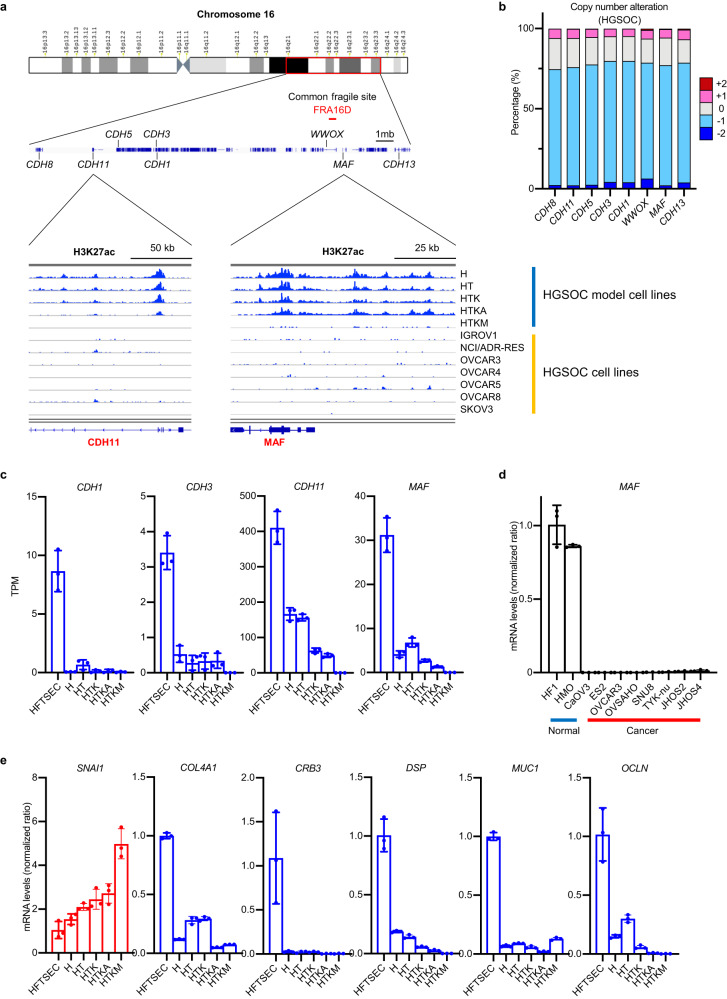
Common chromosomal fragile site in 16D (FRA16D) is transcriptionally suppressed by both genetic and epigenetic mechanisms. **a** ChIP-seq for H3K27ac. Enhancer histone marks around FRA16D are downregulated in ovarian cancer cell lines. **b** CNAs of genes around FRA16D in HGSOC samples. Loss of heterozygosity is frequently observed. Each sample was segregated according to its CNA status: amplification (CNA = +2); gain (CNA = +1); duplicate (CNA = 0); deletion (CNA = −1); and deep deletion (CNA = −2). Data are sourced from the TCGA project of HGSOC. **c** mRNA expression levels (RNA-seq, *n* = 3) of CDH family genes and MAF genes around FRA16D are decreased in HGSOC model cells. Error bars represent the mean ± standard deviation (SD). **d** mRNA expression levels (RT‒qPCR; *n* = 3) of the *MAF* gene are downregulated in HGSOC cell lines. HF1 (immortalized human fallopian tube secretory epithelial cells) and HMO (immortalized human endometrioid cells) are normal control samples. Error bars represent the mean ± SD. **e** mRNA expression levels (RT‒qPCR, *n* = 3) of epithelial-mesenchymal transition (EMT)-related genes. Upregulation of *SNAI1*, a mesenchymal marker, and downregulation of epithelial markers, including *COL4A1*, *CRB3*, *DSP*, *MUC1*, and *OCLN*, are observed during oncogenic transformation. Error bars represent the mean ± standard deviation (SD).

Cadherin family genes encode calcium-dependent cell-cell adhesion glycoproteins. Among them, *CDH1* (E-cadherin), an important EMT-related gene whose downregulation disrupts epithelial integrity, is typically observed at the initiation of EMT^34^. Eight cadherin family genes (*CDH1*, *CDH3*, *CDH5*, *CDH8*, *CDH11*, *CDH13*, *CDH15*, and *CDH16*) are located in the long arm of chromosome 16, forming a cadherin cluster region. When we observed enhancer histone marks in this characteristic genomic region, H3K27ac modification disappeared during the oncogenic transformation of HGSOC (Fig. 5a), indicating that epigenetic silencing of the cadherin cluster region is a causal mechanism of EMT.

The genomic region around the *WWOX* gene on chromosome 16 is termed the common chromosomal fragile site in 16D (FRA16D), in which LOH frequently occurs in HGSOCs (Fig. 5b)^35,36^. Interestingly, this FRA16D site is located in the cadherin cluster region. Therefore, cadherin cluster genes suffer from both LOH and epigenetic silencing during HGSOC initiation, decreasing their mRNA expression levels. In addition, the *MAF* gene, an AP-1 complex component with a potential antagonistic effect on the JUN and FOS family proteins, is located next to *WWOX*. The mRNA expression of *MAF* and cadherin genes (*CDH1*, *CDH3*, and *CDH11*) was highly downregulated in both ovarian cancer cell lines and tissues (Fig. 5c, d, Supplementary Fig. 3a). In addition, *MAF* mRNA expression was highly correlated with *DAB2* mRNA expression in HGSOC tissues, suggesting potential crosstalk between the AP-1 complex and the GATA6-DAB2 axis (Supplementary Fig. 3b).

To confirm that EMT was induced during oncogenic transformation in HGSOC model cells, we conducted RT‒qPCR analysis to assess the expression levels of specific markers. As expected, we observed the upregulation of *SNAI1*, a mesenchymal marker, and the downregulation of epithelial markers, including *COL4A1*, *CRB3*, *DSP*, *GSK3B*, *MUC1*, *OCLN*, and *CDH1* (Fig. 5e, Supplementary Fig. 3c).

**Fig. 6:**
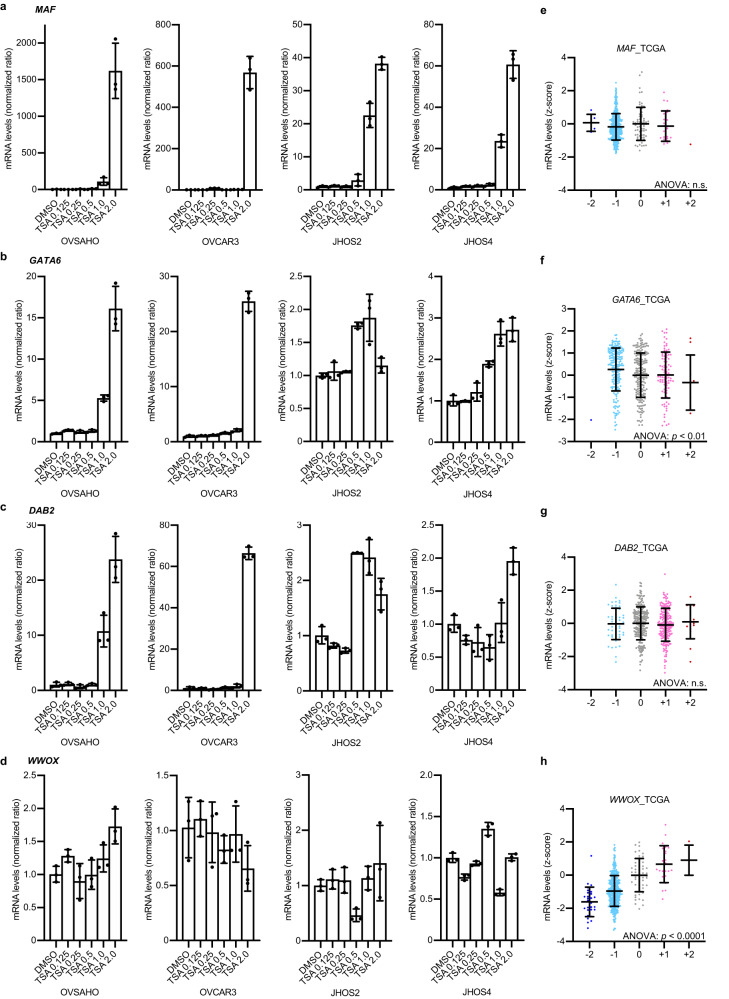
The gene expression of MAF, GATA6 and DAB2 is epigenetically suppressed in HGOSCs. **a–d** mRNA expression levels (RT‒qPCR; *n* = 3) of *MAF*, *GATA6*, *DAB2* and *WWOX* genes upon trichostatin A (TSA) treatment in OVSAHO, OVCAR3, JHOS2 and JHOS4 cells. TSA treatment upregulated the mRNA expression of *MAF*, *GATA6* and *DAB2*. In contrast, the mRNA expression levels of *WWOX* were less affected by TSA treatment. Error bars represent the mean ± standard deviation (SD). **e–h** mRNA expression levels and copy number alteration (CNA) of *MAF*, *GATA6*, *DAB2* and *WWOX* genes were plotted using TCGA HGSOC data (*n* = 489). The mRNA expression of *MAF*, *GATA6* and *DAB2* was less affected by CNA. In contrast, the mRNA expression levels of WWOX were highly correlated with CNA. Each sample was segregated according to its CNA status: amplification (CNA = +2); gain (CNA = +1); duplicate (CNA = 0); deletion (CNA = −1); and deep deletion (CNA = −2). Error bars represent the mean ± SD. Statistical analysis was performed using one-way ANOVA.

Next, to validate whether the downregulated genes, such as *MAF*, *GATA6*, and *DAB2*, are affected by epigenetic dysregulation, we treated ovarian cancer cell lines with a pan-HDAC inhibitor, TSA, which mechanistically augments transcriptionally active histone modification. The mRNA expression of *MAF*, *GATA6*, and *DAB2* was highly upregulated, whereas that of *WWOX* did not change (Fig. 6a–d). The genomic copy number alteration (CNA) profile showed a positive correlation with *WWOX* mRNA expression, whereas it did not affect the mRNA expression of *MAF* and *DAB2* or resulted in an inverse correlation with *GATA6* (Fig. 6e–h). These results indicate that *MAF*, *GATA6*, and *DAB2* undergo epigenetic silencing, whereas *WWOX* is mainly regulated by genomic alterations.

*MAF* mRNA expression is reportedly suppressed by the long noncoding RNAs *DANCR* and EZH2^37^. Indeed, our HGSOC model cells exhibited upregulated expression of *DANCR* and *EZH2*, as well as that of *HDAC1* and *HDAC2* (Supplementary Fig. 3d). siRNA knockdown of *DANCR* resulted in significant suppression of colony formation in HF1/TP53/KRAS/AKT, HF1/TP53/KRAS/MYC, OVCAR3, and CaOV3 cells. This observation suggests that inhibiting *DANCR* could potentially offer therapeutic advantages in modifying the epigenetic dysregulation associated with HGSOCs (Supplementary Fig. 3e). In addition, TSA treatment suppressed the proliferation of ovarian cancer cell lines; interestingly, it exhibited an even higher cytotoxic effect on HF1/TP53/KRAS/MYC cells than on HF1 cells (Supplementary Fig. 3f). The differential response to TSA between HF1/TP53/KRAS/MYC and HF1 cells underscores the importance of further investigations into the underlying epigenetic mechanisms and potential therapeutic implications for specific cancer subtypes.

Overall, these results suggest that *MAF*, *GATA6*, *DAB2*, and cadherin genes are epigenetically downregulated in HGSOCs, possibly accompanying EMT in the early process of tumorigenesis, and can be potential therapeutic targets for HGSOCs.

### Downregulation of *MAF*, *GATA6*, and *DAB2* is associated with proteasome dysregulation and EMT initiation in HFTSECs

The downregulation of *MAF*, *GATA6*, and *DAB2* might be the preceding event of HGSOC tumorigenesis. Therefore, we performed siRNA knockdown experiments and RNA-seq analysis of these three genes in HF1 cells to investigate the effects of their dysregulation in HFTSECs.

**Fig. 7:**
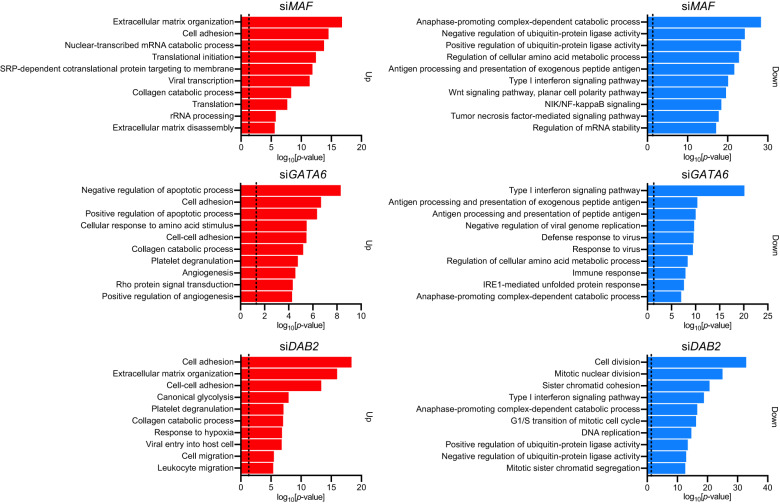
Inhibition of *MAF*, *GATA6* and *DAB2* upregulates epithelial-mesenchymal transition (EMT)-related genes and downregulates proteasome genes. Gene Ontology (GO) analysis of upregulated differentially expressed genes (DEGs) (left, *n* = 500) and downregulated DEGs (right, *n* = 500) obtained from the RNA-seq data of siRNA gene knockdown experiments in HF1 cells. “Cell adhesion” in upregulated DEGs and “Anaphase-promoting complex-dependent catabolic process” in downregulated DEGs were consistently enriched in the gene knockdown experiments of *MAF*, *GATA6* and *DAB2*. Dotted lines indicate the position of *p* = 0.05. upper: siMAF, middle: siGATA6, lower: siDAB2.

GO analysis showed that the GO terms “Cell adhesion” and “Extracellular matrix organization” were enriched in the upregulated DEGs (Fig. 7). This finding may seem contradictory to the GO analysis presented in Fig. 1c, where the GO terms of “cell adhesion” and “extracellular matrix organization” were enriched in the downregulated DEGs during oncogenic transformation. However, upon closer examination of individual DEGs, we found the downregulation of EMT-related epithelial marker genes such as *CDH1* and *COL4A1* (Fig. 1c) and the upregulation of EMT-related mesenchymal marker genes such as *CDH2*, *FN1*, *TGFBI*, *COL1A1*, *COL1A2*, and *MMP2*, as well as poor prognostic factors of ovarian carcinoma, such as *KRT7* and *KRT19* (Fig. 7, Supplementary Fig. 4a–c)^38^. These results collectively support the hypothesis that downregulation of *MAF*, *GATA6*, and *DAB2* triggers EMT and results in the malignant features of HGSOCs.

However, downregulated DEGs were enriched in the GO terms “Anaphase-promoting complex-dependent catabolic process” and “Ubiquitin-protein ligase activity,” which mainly consist of proteasomal regulation-related genes. Among them, *PSMB8* and *PSMB9*, which are classified into a subgroup named “immunoproteasome,” were consistently downregulated upon knockdown of *MAF*, *GATA6*, and *DAB2*, and lower expression of these genes was associated with poor prognosis in patients with HGSOC (Fig. 7, Supplementary Fig. 4d, e).

Immunoproteasomes play important roles in generating HLA peptides for immune cell activation, and their downregulation results in impaired antigen presentation, such that immune cells cannot fully defend against cancer cells^39,40^. As immune escape is another hallmark of cancer, it is reasonable for HGSOCs to downregulate immunoproteasome genes during the early stages of tumorigenesis.

In addition, the inhibition of proteasome activity results in c-Jun activation and EMT initiation, consistent with our results demonstrating the dysregulation of the AP-1 complex in the early stages of HGSOC tumorigenesis^41–43^. Therefore, the downregulation of *MAF*, *GATA6*, and *DAB2* may be associated with dysregulated proteasomal function, resulting in EMT and the immune escape of cancer cells.

The treatment of HF1 cells with carfilzomib, a selective proteasome inhibitor, resulted in significantly downregulated mRNA expression of *MAF* and *GATA6*, indicating a positive correlation between their activities (Supplementary Fig. 5a). In addition, carfilzomib treatment upregulated chemokine gene (*CXCL1*, *CXCL2*, *CXCL3*, *CXCL5*, and *CXCL8*) expression, which causes immune evasion (Supplementary Fig. 5b)^44^. These data suggest that proteasome inhibition occurs during the early stages of HGSOC tumorigenesis, possibly establishing a tumor immune system.

However, carfilzomib treatment upregulated *MAF* mRNA expression in HF1/TP53/KRAS/MYC cells, which was in contrast to the reaction observed in HF1 cells (Supplementary Fig. 5a). Furthermore, carfilzomib exhibited strong cytotoxic effects in HGSOC cell lines, which were lower in HF1 cells than in HF1/TP53/KRAS/MYC cells (Supplementary Fig. 5c). These data suggest that proteasome activity is suppressed in the early stages of tumorigenesis to promote EMT and immune escape, and they gradually activate toward late-stage HGSOCs. These results indicate that proteasome inhibitors potentially exert both pro- and antitumor effects, which may explain the emergence of chemoresistance to proteasome inhibitors in clinical situations.

### An MEK inhibitor reverses the dysregulated transcriptome in HGSOCs

MAF antagonizes KRAS V12-induced cell proliferation, and DAB2 inhibits Ras activation by interacting with Ras GTPase-activating protein^45,46^. Thus, it is deduced that MAF downregulation and the GATA6-DAB2 axis redundantly cause aberrant Ras activation and proteasomal dysregulation. Therefore, we hypothesized that the inhibition of Ras signaling mitigates oncogenic effects by downregulating *MAF*, *GATA6*, and *DAB2*.

**Fig. 8:**
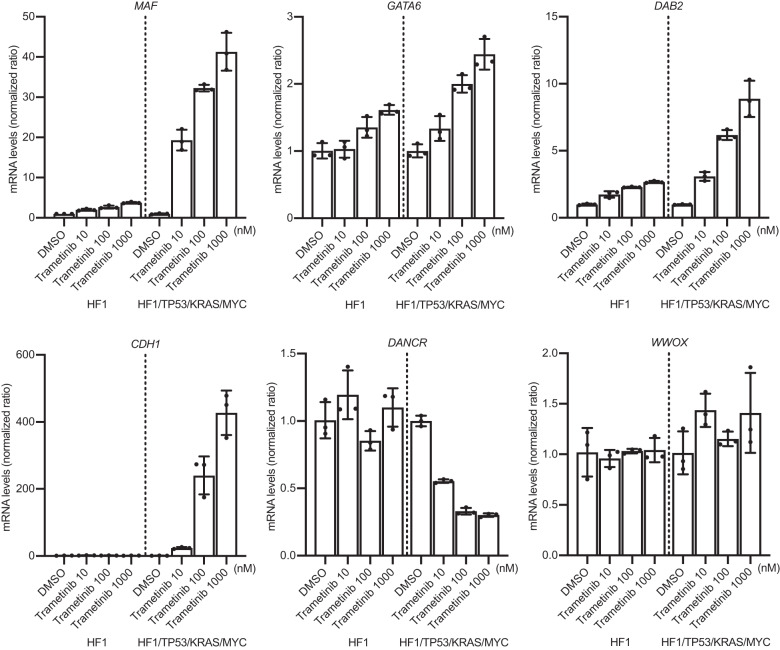
A MEK inhibitor reverses early oncogenic alterations in HGSOC model cells. mRNA expression levels (RT‒qPCR; *n* = 3) of *MAF*, *GATA6*, *DAB2*, *CDH1*, *DANCR* and *WWOX* genes upon trametinib treatment in HF1 and HF1/TP53/KRAS/MYC cells. Trametinib treatment upregulates the mRNA expression levels of *MAF*, *GATA6*, *DAB2* and *CDH1*. These effects are higher in HF1/TP53/KRAS/MYC cells than in HF1 cells. In contrast, the mRNA expression levels of DANCR were decreased only in HF1/TP53/KRAS/MYC cells, and those of *WWOX* were less affected by trametinib treatment. Error bars represent the mean ± standard deviation (SD).

To confirm this, we treated HF1 and HF1/TP53/KRAS/MYC cells with trametinib, a clinically applicable MEK inhibitor. Trametinib treatment significantly increased *MAF*, *GATA6*, and *DAB2* mRNA expression levels. Importantly, the effect of trametinib on the upregulation of these genes was greater in HF1/TP53/KRAS/MYC cells than in HF1 cells, suggesting that an MEK inhibitor effectively reversed the oncogenic transformation of HGSOCs. Similarly, *CDH1* mRNA expression was highly increased upon trametinib treatment in HF1/TP53/KRAS/MYC cells. *DANCR*, an *MAF* suppressor, was downregulated only in HF1/TP53/KRAS/MYC cells. In contrast, WWOX expression did not change, indicating *that MAF* was selectively upregulated by the MEK inhibitor (Fig. 8). The aberrant mRNA expression of immunoproteasomes (*PSMB8* and *PSMB9*) and chemokines (*CXCL1*, *CXCL2*, *CXCL3*, *CXCL5*, and *CXCL8*) was also recovered by trametinib in HF1/TP53/KRAS/MYC cells (Supplementary Fig. 6a).

Additionally, SNU8, an ovarian cancer cell line with the *KRAS V12* mutation, exhibited similar trends in mRNA expression following trametinib treatment (Supplementary Fig. 6b, c). Importantly, trametinib treatment showed higher cytotoxic effects in SNU8 and HF1/TP53/KRAS/MYC cells than in HF1 cells (Supplementary Fig. 6d). These results suggest that the epigenetic suppression of *MAF*, *GATA6*, and *DAB2* expression was rescued by trametinib treatment, accompanied by EMT restoration, proteasomal dysregulation, and immune evasion in HGSOCs.

## Discussion

In this study, integrative epigenomic analysis revealed that the DNA-binding activities of JUN and FOS family proteins were elevated, whereas those of GATA family proteins were alleviated in the early stages of HGSOC tumorigenesis.

The AP-1 complex is a heterodimer comprising members of the JUN, FOS, ATF, and MAF family proteins. Our analysis revealed that the mRNA expression of *MAF* was substantially downregulated by both genomic and epigenetic alterations in HGSOCs. Theoretically, *MAF* downregulation increases the chance of DNA binding for JUN or FOS family proteins, leading to their relatively high cellular influence^47^. This effect may contribute to the increased DNA-binding activities of JUN and FOS family proteins, which induce EMT and cell proliferation in malignant tumors^32^. The activation of Ras and AP-1 signaling suppresses *MAF* mRNA expression^48^. In addition, MAF antagonizes Ras-driven tumor cell proliferation^45^. These reports suggest that *MAF* downregulation has survival advantages for HGSOC development, possibly due to the activation of Ras signaling.

In GATA family proteins, we identified *GATA6* as a potential tumor suppressor gene whose expression is suppressed in HGSOCs. GATA6 downregulates the mRNA expression of *DAB2*, a known tumor suppressor gene in ovarian cancer^30^. Both *GATA6* and *DAB2* were downregulated in HGSOCs. DAB2 functionally antagonizes Ras signaling by interacting with a Ras GTPase-activating protein^46^. Furthermore, the mRNA expression of *MAF* and *DAB2* was reasonably correlated in HGSOCs, suggesting potential crosstalk between MAF and the GATA6–DAB2 axis.

The downregulation of *MAF*, *GATA6*, and *DAB2* was consistently associated with EMT initiation and decreased proteasome expression in immortalized HFTSECs. Proteasome inhibition induces EMT and AP-1 activation^41–43^. These findings collectively suggest that the suppression of *MAF*, *GATA6*, and *DAB2* causes the activation of Ras and AP-1 signaling, accompanied by EMT in HGSOCs^49^. In addition, our analysis revealed that immunoproteasome genes (*PSMB8* and *PSMB9*) were downregulated, and chemokine genes (*CXCL1*, *CXCL2*, *CXCL3*, *CXCL5*, and *CXCL8*) were upregulated by these oncogenic alterations. As the dysregulation of immunoproteasomes and chemokines promotes immune evasion of cancer cells, suppression of *MAF*, *GATA6*, and *DAB2* may contribute to the maintenance of the tumor microenvironment, which is advantageous for EMT and immune escape in the early stages of tumorigenesis^39,40^.

However, proteasomal regulation could be a double-edged sword for tumor development. Advanced tumors show enhanced proteasomal activity to degrade aberrantly overexpressed or misfolded proteins. Proteasome inhibitors cause cytotoxic effects in cancer cells and are currently in clinical trials^50^. However, chemoresistance severely limits the clinical applications of proteasome inhibitors. Our results showed that proteasome inhibition contributes to EMT and immune escape, which may partly explain the chemoresistance to proteasome inhibitors.

In the present study, to address this problem, we investigated the effects of an MEK inhibitor in HGSOC model cells because MAF and DAB2 are known antagonists of Ras signaling. The results revealed that the MEK inhibitor successfully reversed the downregulation of *MAF*, *GATA6*, and *DAB2* as well as EMT and proteasomal dysregulation. Given that chemoresistance to proteasome inhibitors is due to the survival of stem cell-like cancer cells that can undergo EMT^51^, MEK inhibitors may overcome recurrent tumors induced by proteasome inhibitors (Supplementary Fig. 7).

There are a few limitations to the present study. The main analyses and experiments were conducted in silico and in vitro. Therefore, our findings must be verified in vivo in future studies. The clinical applicability of drugs targeting the AP-1 complex and GATA family proteins has not yet been established. Therefore, the development of potent molecular-targeted drugs for dysregulated transcription factors is needed.

Nevertheless, the present study provides a characteristic profile of early epigenetic changes in HGSOC tumorigenesis and identifies targetable molecules and pathways, such as lincRNA *DANCR*, Ras signaling, and proteasomal dysregulation. The results obtained in this study offer useful information for cancer epigenomic studies and may contribute to the prevention of HGSOC tumorigenesis.

In this study, we performed an integrative epigenomic analysis of a series of HGSOC model cells derived from HFTSECs to identify the early epigenetic changes in HGSOC tumorigenesis and therapeutic target molecules and pathways. Our findings revealed that the dysregulation of the AP-1 complex and GATA6-DAB2 axis in HGSOCs triggers EMT and proteasomal dysregulation. Furthermore, an MEK inhibitor successfully reversed these oncogenic alterations, suggesting that inhibitors of Ras signaling are clinically effective in a subgroup of patients with HGSOC. Future validation of these results in large samples may help prevent tumor formation and develop novel therapies for HGSOC. These results also suggest the importance of considering not only genomic abnormalities but also epigenomic abnormalities in the pathogenesis of HGSOC.

### Supplementary information

Supplementary information

Supplementary Table 3

## Data Availability

Raw sequencing data generated for RNA-seq, ATAC-seq, and ChIP-seq have been deposited in the DDBJ database under accession number DRA015121.
