# Supplementary material for: Integrative analysis reveals early epigenetic alterations in high-grade serous ovarian carcinomas
Source: Exp Mol Med. 2023 Oct 2;55(10):2205–19. doi: 10.1038/s12276-023-01090-1 (PMC10618212; doi:10.1038/s12276-023-01090-1)
Supplement: Supplementary file 1 — Supplementary information [file 12276_2023_1090_MOESM1_ESM.pdf]

Supplementary information for
Integrative analysis reveals early epigenetic alterations in high-grade serous ovarian carcinomas

Hidenori Machino, Ai Dozen, Mariko Konaka, Masaaki Komatsu, Kohei Nakamura, Noriko Ikawa, Kanto Shozu, Ken Asada, Syuzo Kaneko, Hiroshi Yoshida, Tomoyuki Kato, Kentaro Nakayama, Vassiliki Saloura, Satoru Kyo, and Ryuji Hamamoto

The file contains
Supplementary Tables 1 – 2
Supplementary Figures 1 – 7

Supplementary Table 1 Information of certified cell lines.

Name	Certification institution	*BRCA1*	*BRCA2*	Tested method	DNA profile or characteristics
293T	ATCC	Not assessed	Not assessed	STR	Amelogenin: X CSF1PO: 11, 12 D13S317: 12, 14 D16S539: 9, 13 D5S818: 8, 9 D7S820: 11 THO1: 7, 9.3 TPOX: 11 vWA: 16, 19
CaOV3	ATCC	Wild type	Wild type	STR	Amelogenin: X CSF1PO: 10, 13 D13S317: 12 D16S539: 9 D5S818: 12 D7S820: 10 THO1: 7 TPOX: 8, 10 vWA: 16, 18
ES2	ATCC	Wild type	Wild type	STR	Amelogenin: X CSF1PO: 10 D13S317: 11 D16S539: 11, 13 D5S818: 11, 13 D7S820: 11 THO1: 9.3 TPOX: 8, 12 vWA: 16, 17
JHOS-2	RIKEN CELL BANK	M1? (Driver)	Wild type	STR	Amelogenin: X CSF1PO: 11 D13S317: 8, 9 D16S539: 13 D21S11: 31, 31.2 D5S818: 10 D7S820: 11 THO1: 6, 7 TPOX: 8, 11 vWA: 14, 17
JHOS-4	RIKEN CELL BANK	Y1202Qfs *12 (Driver)	Wild type	STR	Amelogenin: X CSF1PO: 10 D13S317: 11 D16S539: 9, 10 D21S11: 29, 31.2 D5S818: 11 D7S820: 10 THO1: 7 TPOX: 8, 12 vWA: 14
OVCAR3	RIKEN CELL BANK	Wild type	HOMDEL (Driver)	STR	Amelogenin: X CSF1PO: 11, 12 D13S317: 12 D16S539: 12 D5S818: 11, 12 D7S820: 10 THO1: 9, 9.3 TPOX: 8 vWA: 17
KURAMOCHI	JCRB	HOMDEL (Driver)	R2318* (Driver)	STR	Amelogenin: X CSF1PO: 11, 12 D13S317: 9,12 D16S539: 10 D5S818: 12 D7S820: 10, 11 THO1: 9 TPOX: 8, 12 vWA: 16, 19
OVSAHO	JCRB	Wild type	HOMDEL (Driver)	STR	Amelogenin: X CSF1PO: 10, 12 D13S317: 8 D16S539: 9 D5S818: 12, 13 D7S820: 8, 10 THO1: 6 TPOX: 8, 11 vWA: 14
RMUGS	JCRB	Wild type	Wild type	STR	Amelogenin: X CSF1PO: 12 D13S317: 10, 11 D16S539: 10, 11 D5S818: 10, 14 D7S820: 12 THO1: 9 TPOX: 8, 11 vWA: 14, 16
TYK-nu	JCRB	Wild type	Wild type	STR	Amelogenin: X CSF1PO: 12 D13S317: 10, 11 D16S539: 9, 10 D5S818: 12, 13 D7S820: 10 THO1: 9 TPOX: 9, 11 vWA: 14, 16
SNU8	KCLB	Wild type	Wild type	STR	Amelogenin: X CSF1PO: 9, 12 D13S317: 8, 9 D3S1358: 14, 16 D5S818: 11, 12 D7S820: 8, 12 FGA: 20, 26 THO1: 7, 9 TPOX: 11 vWA: 17

ATCC; American Type Culture Collection
JCRB; Japanese Collection of Research Bioresources
KCLB; Korean Cell Line Bank
BRCA1 and *BRCA2* gene profiles were sourced from Cancer Cell Line Encyclopedia (CCLE).

Supplementary Table 2 Information of primers.

Primer names	Purpose	Direction	Sequence (5' -> 3' direction)
GAPDH	qPCR	Forward	GCAAATTCCATGGCACCGTC
		Reverse	TCGCCCCACTTGATTTTGG
MAF	qPCR	Forward	TATGCCCAGTCCTGCCGCTT
		Reverse	CGCTGCTCGAGCCGTTTTCT
GATA6	qPCR	Forward	GCCACTACCTGTGCAACGCCT
		Reverse	CAATCCAAGCCGCCGTGATGAA
DAB2	qPCR	Forward	CTCTGTCCAGTCCTCACCACAT
		Reverse	GTTCTGAGACGGGAGGAGCAAA
WWOX	qPCR	Forward	TCCTCCAGGATGTTTTGTGCCG
		Reverse	AAGCCAGCATCGCCCAATAGTC
CDH1	qPCR	Forward	GCCTCCTGAAAAGAGAGTGGAAG
		Reverse	TGGCAGTGTCTCTCCAAATCCG
DANCR	qPCR	Forward	GCCACTATGTAGCGGGTTTC
		Reverse	ACCTGCGCTAAGAACTGAGG
CXCL1	qPCR	Forward	AGCTTGCCTCAATCCTGCATCC
		Reverse	TCCTTCAGGAACAGCCACCAGT
CXCL2	qPCR	Forward	AGGGGTTCGCCGTTCTCGGA
		Reverse	CCGCAGGAGCCGGGGATTG
CXCL3	qPCR	Forward	CGCCCAAACCGAAGTCATAG
		Reverse	GCTCCCCTTGTTCAGTATCTTTT
CXCL5	qPCR	Forward	GAGAGCTGCGTTGCGTTTGTTTAC
		Reverse	CCGTTCTTCAGGGAGGCTACCACT
CXCL8	qPCR	Forward	CACTGCGCCAACACAGAAAT
		Reverse	GCCCTCTTCAAAAACTTCTCCAC
PSMB8	qPCR	Forward	CCTTACCTGCTTGGCACCATGT
		Reverse	TTGGAGGCTGCCGACACTGAAA
PSMB9	qPCR	Forward	CGAGAGGACTTGTCTGCACATC
		Reverse	CACCAATGGCAAAAGGCTGTCG
CCND1	qPCR	Forward	CTGGCCATGAACTACCTGGA
		Reverse	GTCACACTTGATCACTCTGG
VEGFA	qPCR	Forward	CAAGACAAGAAAATCCCTGTGG
		Reverse	GCTTGTCACATCTGCAAGTACG
RPL5	qPCR	Forward	CCAAATACAGGATGATAGTTCGTG
		Reverse	TTGGCAGTTCGTGTGCATACGC
RPL6	qPCR	Forward	CCTTGTCAGAGGAATTGGCAGG
		Reverse	GTAACAGTTGCGAGAACCTTCTC
RPL7L1	qPCR	Forward	TGACAAGGTGCGTCTCAGACGA
		Reverse	TCTGCACCAGTAAACTCACGCC
RPL14	qPCR	Forward	GCCGCGAGTAAAAAGGCTCCAG
		Reverse	TGCCTTTGGAGCAGGTGCTTTC
RPL24	qPCR	Forward	CTCGGCAGATAAACTGGACTGTC
		Reverse	GCAAGAGATGCACCAGTAATGGC
RPL26	qPCR	Forward	GGCTAATGGCACAACTGTCCAC
		Reverse	GGCGAGATTTGGCTTTCCGTTC
RPL27	qPCR	Forward	TGGACAAAACTGTCGTCAATAAGG
		Reverse	AGAACCACTTGTTCTTGCCTGTC
RPL34	qPCR	Forward	GACCTAAAGTTCTTATGAGATTGTC
		Reverse	CTGACTCTGTGCTTGTGCCTTC

RPS23	qPCR	Forward	AGGAAGTGTGTAAGGGTCCAGC
		Reverse	CACCAACAGCATGACCTTTGCG
RPS27A	qPCR	Forward	GCAGAGACTGATCTTTGCTGGC
		Reverse	CTTGGGAGTGGTGTAAGACTTCT
SNAI1	qPCR	Forward	TGCCCTCAAGATGCACATCCGA
		Reverse	GGGACAGGAGAAGGGCTTCTC
COL4A1	qPCR	Forward	TGTTGACGGCTTACCTGGAGAC
		Reverse	GGTAGACCAACTCCAGGCTCTC
CRB3	qPCR	Forward	CTTCTGCAAATGAGAATAGCACTG
		Reverse	GACCACGATGATAGCAGTGATGG
DSP	qPCR	Forward	TGACAGACCGCTGGCAAAGGAT
		Reverse	GGCGTTTAGCATCATAGAGCCAC
MUC1	qPCR	Forward	CCTACCATCCTATGAGCGAGTAC
		Reverse	GCTGGGTTTGTGTAAGAGAGGC
OCLN	qPCR	Forward	ATGGCAAAGTGAATGACAAGCGG
		Reverse	CTGTAACGAGGCTGCCTGAAGT
GSK3B	qPCR	Forward	CCGACTAACACCACTGGAAGCT
		Reverse	AGGATGGTAGCCAGAGGTGGAT

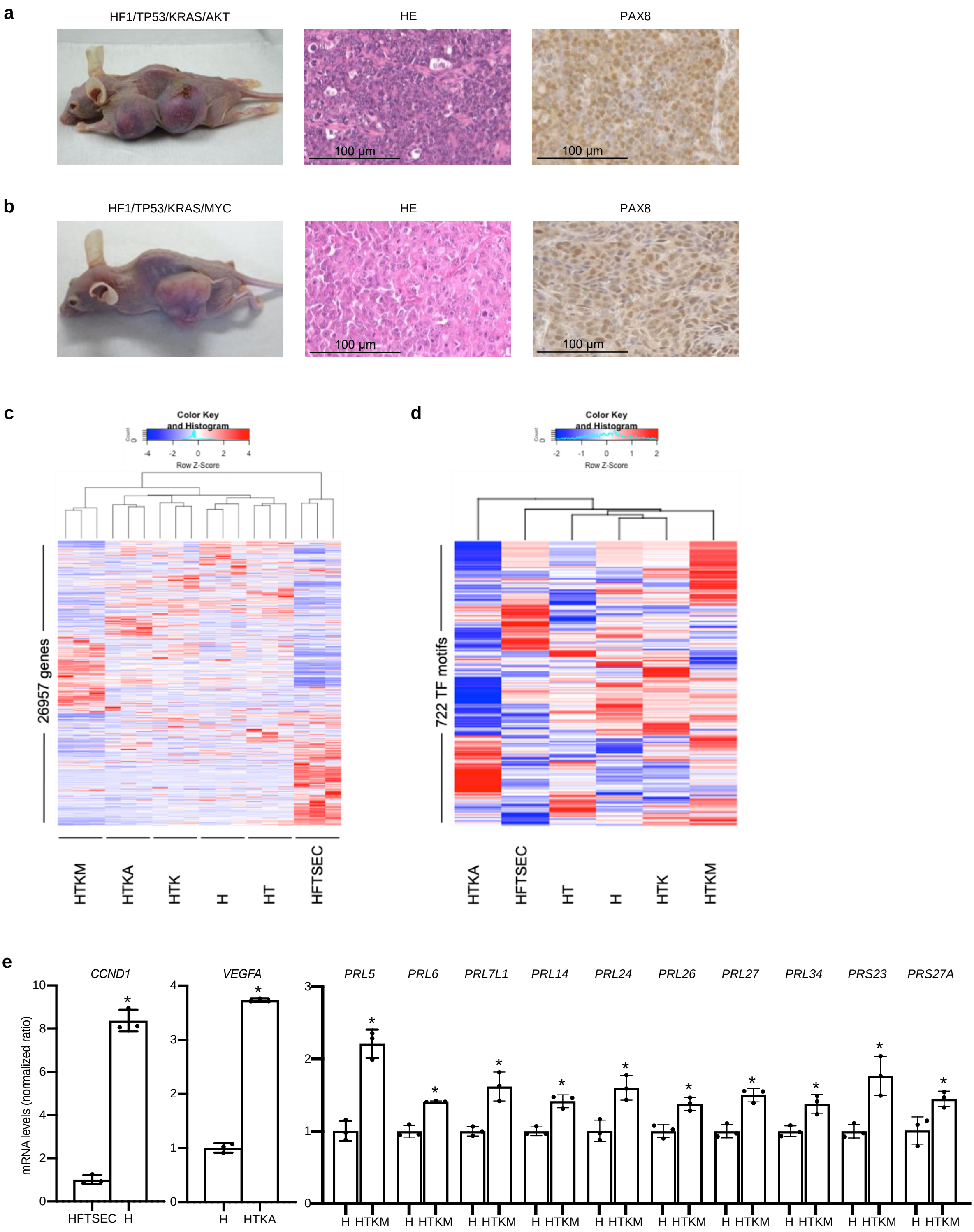
a
HF1/TP53/KRAS/AKT
HE
PAX8
100 μm
100 μm
b
HF1/TP53/KRAS/MYC
HE
PAX8
100 μm
100 μm
c
Color Key and Histogram
Row Z-Score
26957 genes
HTKM
HTKA
HTK
H
HT
HFTSEC
d
Color Key and Histogram
Row Z-Score
722 TF motifs
HTKA
HFTSEC
HT
H
HTK
HTKM
e
mRNA levels (normalized ratio)
CCND1
VEGFA
PRL5
PRL6
PRL7L1
PRL14
PRL24
PRL26
PRL27
PRL34
PRS23
PRS27A
HFTSEC
H
HTKA
HTKM

Supplementary Fig. 1 Mouse xenograft experiments and integrative analysis of stepwise HGSOC model cells. a–b Subcutaneous mouse xenograft experiments using HF1/TP53/KRAS/AKT (a) and HF1/TP53/KRAS/MYC (b) cells, respectively. The confirmation of the HGSOC-like phenotype in the mouse xenograft experiments was carried out by a pathologist who examined the results of hematoxylin eosin (HE) staining and PAX8 staining, which is a positive marker for HGSOC. Scale bars, 100 μm. Detailed information of mouse xenograft experiments is previously described[11]. **c** A heatmap with hierarchical clustering of gene expression levels calculated using RNA-seq. **d** A heatmap with hierarchical clustering of TF motif enrichment scores calculated using ATAC-seq. **e** mRNA expression levels (RT-qPCR; n = 3) of *CCND1*, *VEGFA,* and ribosomal protein genes in the indicated HGSOC model cells. Error bars represent mean ± standard deviation (SD). Statistical analysis was performed using unpaired Student's t-test. * $p < 0.05$.

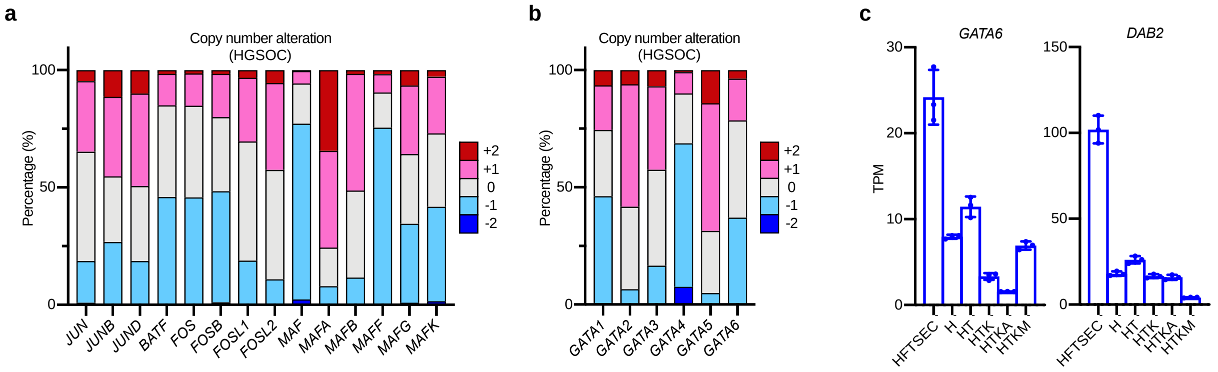

Supplementary Fig. 2 AP-1 family and GATA family genes are dysregulated in HGSOCs. a–b Copy number alteration (CNA) of AP-1 family genes (a) and GATA family genes (b) in HGSOC samples**.** Each sample was segregated according to their CNA status: amplification (CNA = +2); gain (CNA = +1); duplicate (CNA = 0); deletion (CNA = -1); deep deletion (CNA = -2). Data are sourced from TCGA project of HGSOC. **c** mRNA expression levels (RNA-seq, n = 3) of *GATA6* and *DAB2* are downregulated in HGSOC model cells. Error bars represent mean ± standard deviation (SD).

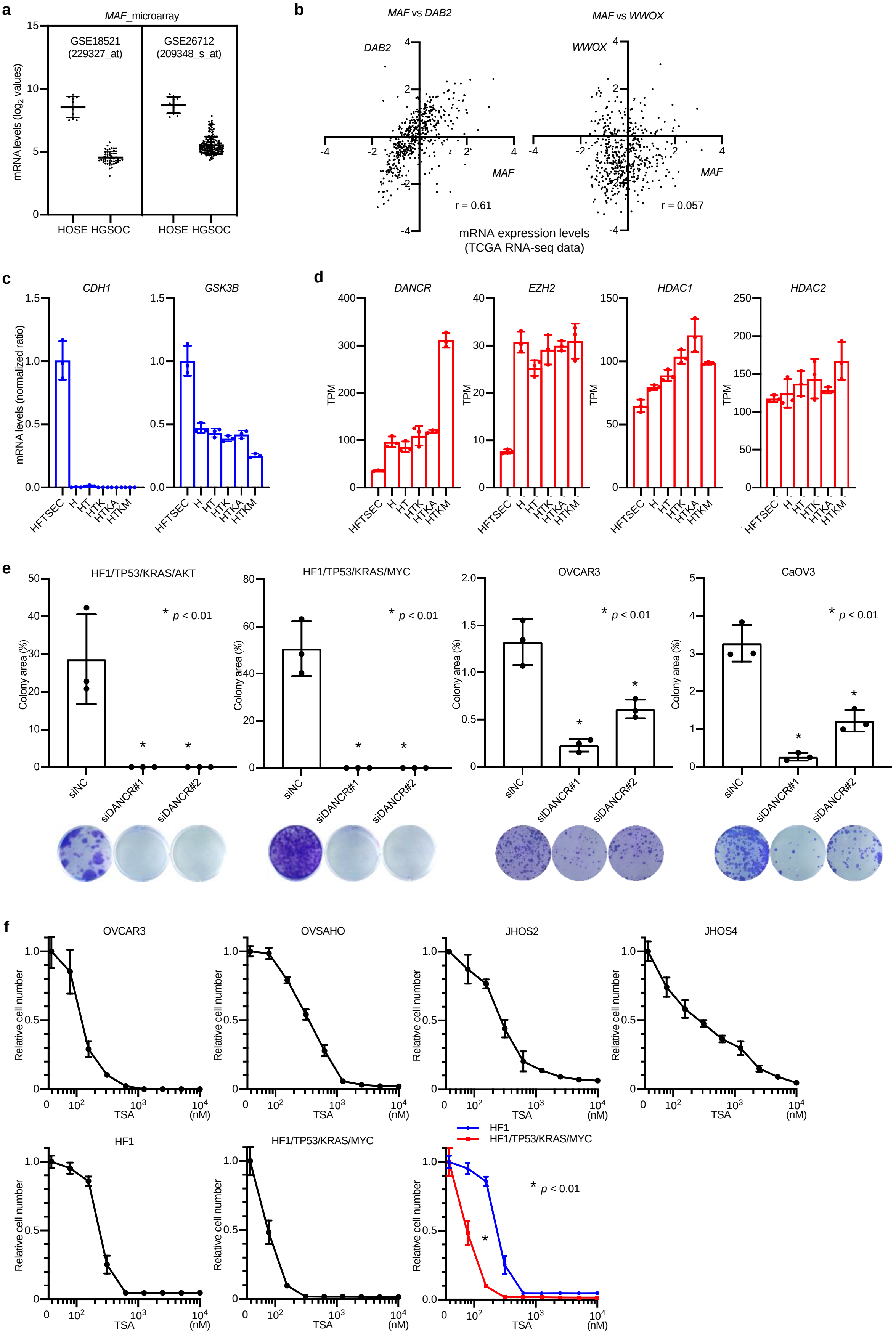
a
MAF_microarray
GSE18521 (229327_at)
GSE26712 (209348_s_at)
mRNA levels (log2 values)
HOSE HGSOC
HOSE HGSOC
b
MAF vs DAB2
MAF vs WWOX
DAB2
WWOX
MAF
MAF
r = 0.61
r = 0.057
mRNA expression levels
(TCGA RNA-seq data)
c
CDH1
GSK3B
mRNA levels (normalized ratio)
HFTSEC H HT HTK HTKA HTKM
d
DANCR
EZH2
HDAC1
HDAC2
TPM
HFTSEC H HT HTK HTKA HTKM
e
HF1/TP53/KRAS/AKT
HF1/TP53/KRAS/MYC
OVCAR3
CaOV3
Colony area (%)
* p < 0.01
siNC
siDANCR#1
siDANCR#2
f
OVCAR3
OVSAHO
JHOS2
JHOS4
HF1
HF1/TP53/KRAS/MYC
Relative cell number
TSA
(nM)
HF1
HF1/TP53/KRAS/MYC
* p < 0.01

Supplementary Fig. 3 Integrative analysis identifies targetable genes in HGSOCs. a mRNA expression levels of *MAF* in two independent microarray datasets. GSE18521 and GSE26712 contain microarray data of human ovarian surface epithelial cell (HOSE) samples and HGSOC samples [GSE18521(HOSE: n = 10, HGSOC: n = 53) and GSE26712 (HOSE: n = 10, HGSOC: n = 185)]. Statistical analysis was performed using unpaired Student's t-test. Error bars represent mean ± standard deviation (SD). **b** mRNA expression levels of *MAF* are plotted against those of *DAB2* and *WWOX* (n = 489). The data were analyzed using Pearson's correlation coefficients. **c** mRNA expression levels (RT-qPCR, n = 3) of epithelial-mesenchymal transition (EMT)-related genes. Downregulation of epithelial markers, including *CDH1* and *GSK3B*, are observed during oncogenic transformation. Error bars represent mean ± standard deviation (SD). **d** mRNA expression levels (RNA-seq, n = 3) of *DANCR*, *EZH2*, *HDAC1* and *HDAC2* are upregulated in HGSOC model cells. Error bars represent mean ± SD. **e** Colony formation assay with *DANCR* siRNA knockdown experiments in HF1/TP53/KRAS/AKT, HF1/TP53/KRAS/MYC, OVCAR3, and CaOV3 cells. Error bars represent mean ± SD of three biological replicates. Statistical analysis was performed using unpaired Student's t-test. **f** Cell viability assay with TSA treatment. The cytotoxic effects of TSA are amplified in tumorigenic HGSOC model cells. Relative cell number shows the relative ratio between the number of cells in the absence of the inhibitors and the number of cells in the presence of various concentrations of TSA. Error bars represent mean ± SD of three biological replicates.

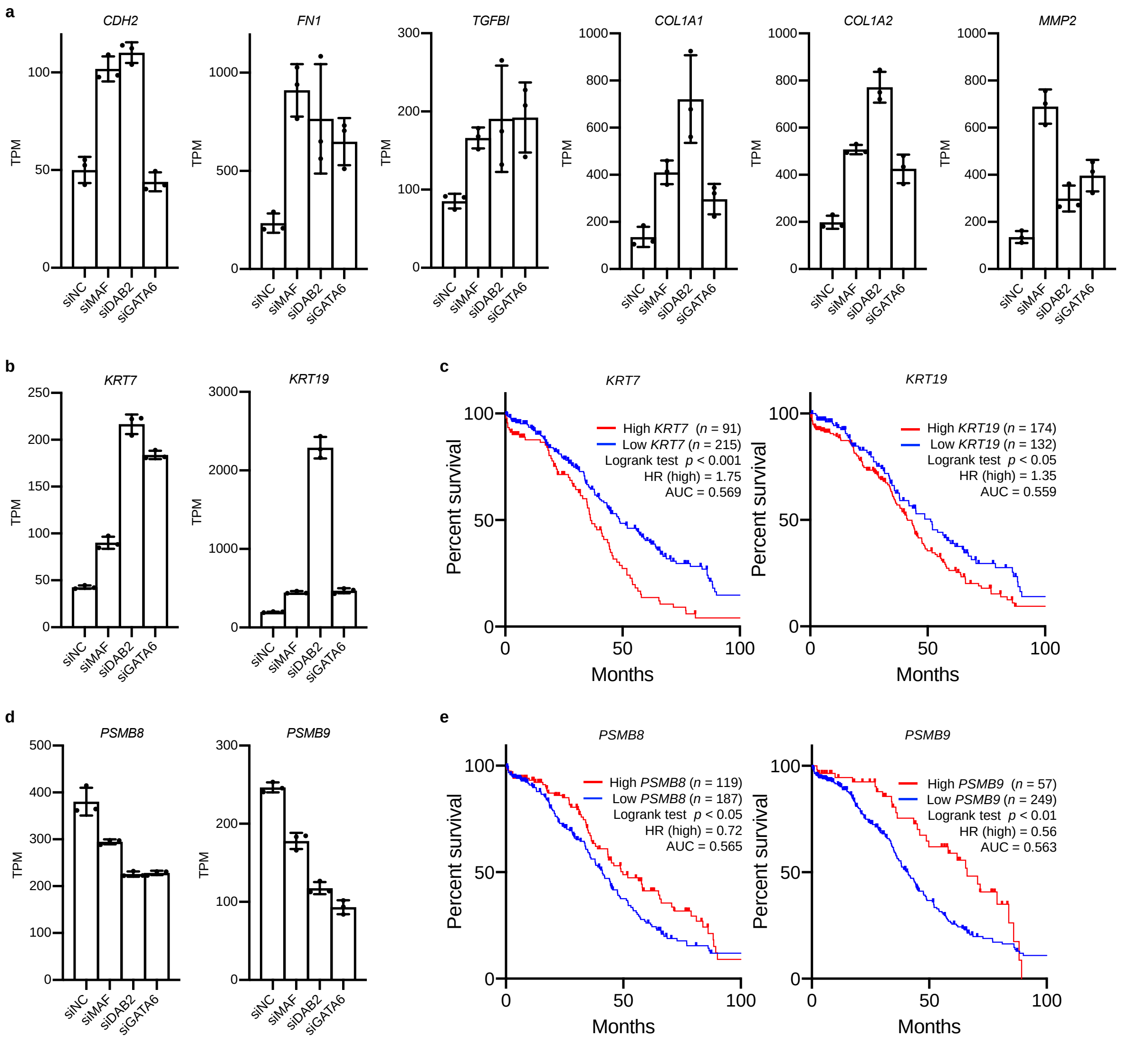

Supplementary Fig. 4 Inhibition of *MAF*, *GATA6* and *DAB2* upregulates epithelial-mesenchymal transition (EMT)-related genes and poor prognostic genes of HGOSCs. a mRNA expression levels (RNA-seq; n = 3) of EMT-related genes upon siRNA knockdown of *MAF*, *GATA6* and *DAB2* in HF1 cells. Inhibition of *MAF*, *GATA6* and *DAB2* upregulates EMT-related genes. Error bars represent mean ± standard deviation (SD). **b** mRNA expression levels (RNA-seq; n = 3) of *KRT7* and *KRT19* genes upon siRNA knockdown of *MAF*, *GATA6* and *DAB2* in HF1 cells. Error bars represent mean ± SD. **c** Kaplan-Meier survival curves classified by high or low *KRT7* or *KRT19* mRNA expression levels in the TCGA HGSOC cohort. High *KRT7* and high *KRT19* group exhibits poor overall survival. **d** mRNA expression levels (RNA-seq; n = 3) of *PSMB8* and *PSMB9* genes upon siRNA knockdown of *MAF*, *GATA6* and *DAB2* in HF1 cells. Error bars represent mean ± SD. **e** Kaplan-Meier survival curves classified by high or low *PSMB8* or *PSMB9* mRNA expression levels in the TCGA HGSOC cohort. Low *PSMB8* and Low *PSMB9* group exhibits poor overall survival.

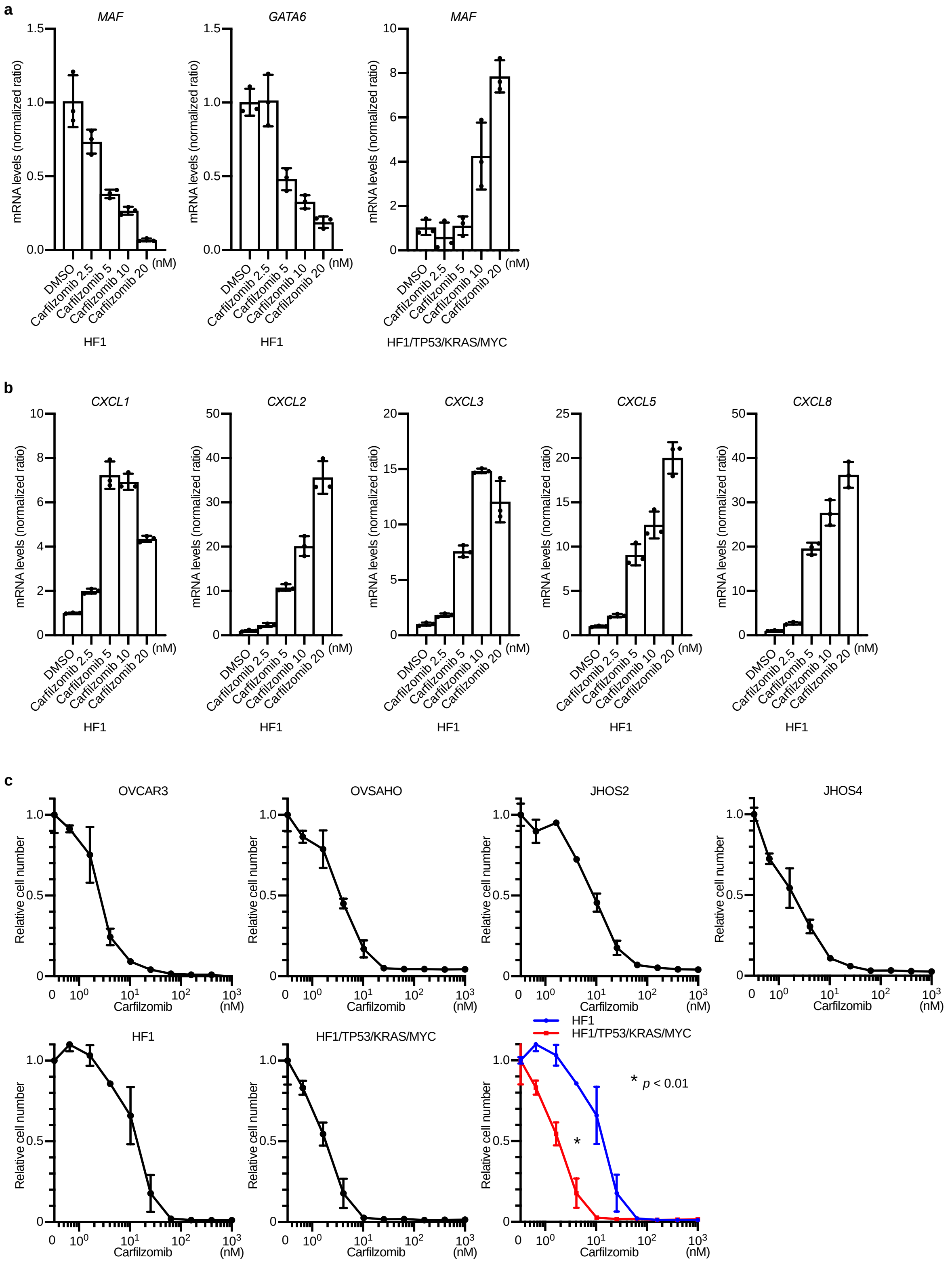
a
MAF
GATA6
MAF
mRNA levels (normalized ratio)
DMSO
Carfilzomib 2.5
Carfilzomib 5
Carfilzomib 10
Carfilzomib 20
(nM)
HF1
HF1/TP53/KRAS/MYC
b
CXCL1
CXCL2
CXCL3
CXCL5
CXCL8
c
OVCAR3
OVSAHO
JHOS2
JHOS4
HF1
HF1/TP53/KRAS/MYC
Relative cell number
Carfilzomib
(nM)
* p < 0.01

Supplementary Fig. 5 A proteasome inhibitor causes both of early oncogenic alterations and tumor suppressive effects. a mRNA expression levels (RT-qPCR; n = 3) of *MAF*, *GATA6* upon carfilzomib treatment in HF1 and HF1/TP53/KRAS/MYC cells. Inhibition of proteasome imitate the early oncogenic alterations of *MAF* and *GATA6* downregulation in HF1 cells, whereas it recovers *MAF* expression in HF1/TP53/KRAS/MYC cells. Error bars represent mean ± standard deviation (SD). **b** mRNA expression levels (RT-qPCR; n = 3) of chemokine genes are upregulated upon carfilzomib treatment in HF1 cells. Error bars represent mean ± SD. **c** Cell viability assay with carfilzomib treatment. The cytotoxic effects of carfilzomib are amplified in HGSOC model cells. Relative cell number shows the relative ratio between the number of cells in the absence of the inhibitors and the number of cells in the presence of various concentrations of carfilzomib. Error bars represent mean ± SD of three biological replicates.

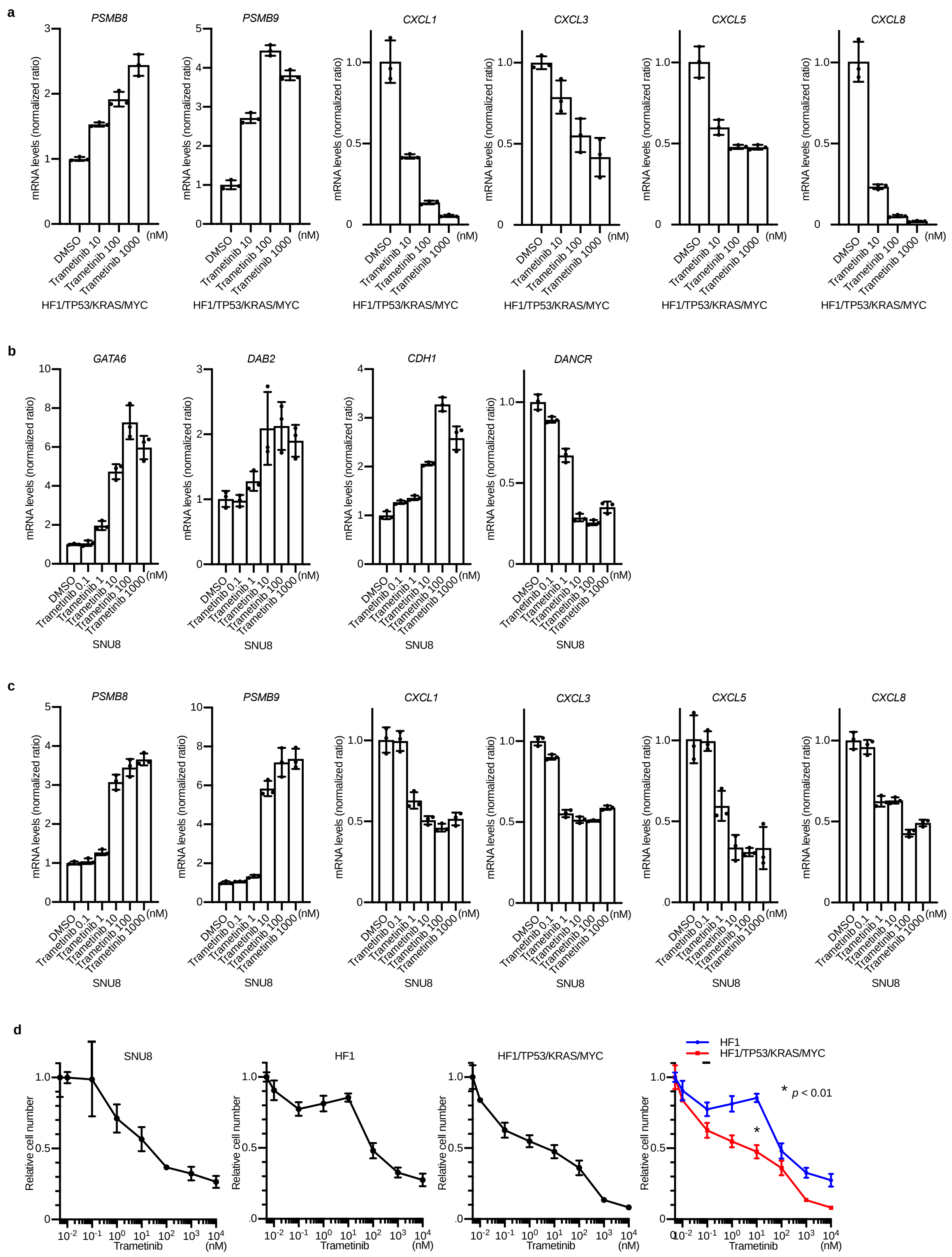
a
PSMB8
PSMB9
CXCL1
CXCL3
CXCL5
CXCL8
mRNA levels (normalized ratio)
DMSO
Trametinib 10
Trametinib 100
Trametinib 1000
(nM)
HF1/TP53/KRAS/MYC
b
GATA6
DAB2
CDH1
DANCR
Trametinib 0.1
Trametinib 1
SNU8
c
d
SNU8
HF1
HF1/TP53/KRAS/MYC
Relative cell number
Trametinib
HF1
* p < 0.01

Supplementary Fig. 6 A MEK inhibitor reverses early oncogenic alterations in HGSOCs. a mRNA expression levels (RT-qPCR; n = 3) of immunoproteasome and chemokine genes upon trametinib treatment in HF1/TP53/KRAS/MYC cells. Error bars represent mean ± standard deviation (SD). **b,c** mRNA expression levels (RT-qPCR; n = 3) of dysregulated genes in HGSOCs are recovered upon carfilzomib treatment in SNU8 cells. Error bars represent mean ± SD. **d** Cell viability assay with trametinib treatment. The cytotoxic effects of trametinib are amplified in HGSOC model cells. Relative cell number shows the relative ratio between the number of cells in the absence of the inhibitors and the number of cells in the presence of various concentrations of carfilzomib. Error bars represent mean ± SD of three biological replicates.

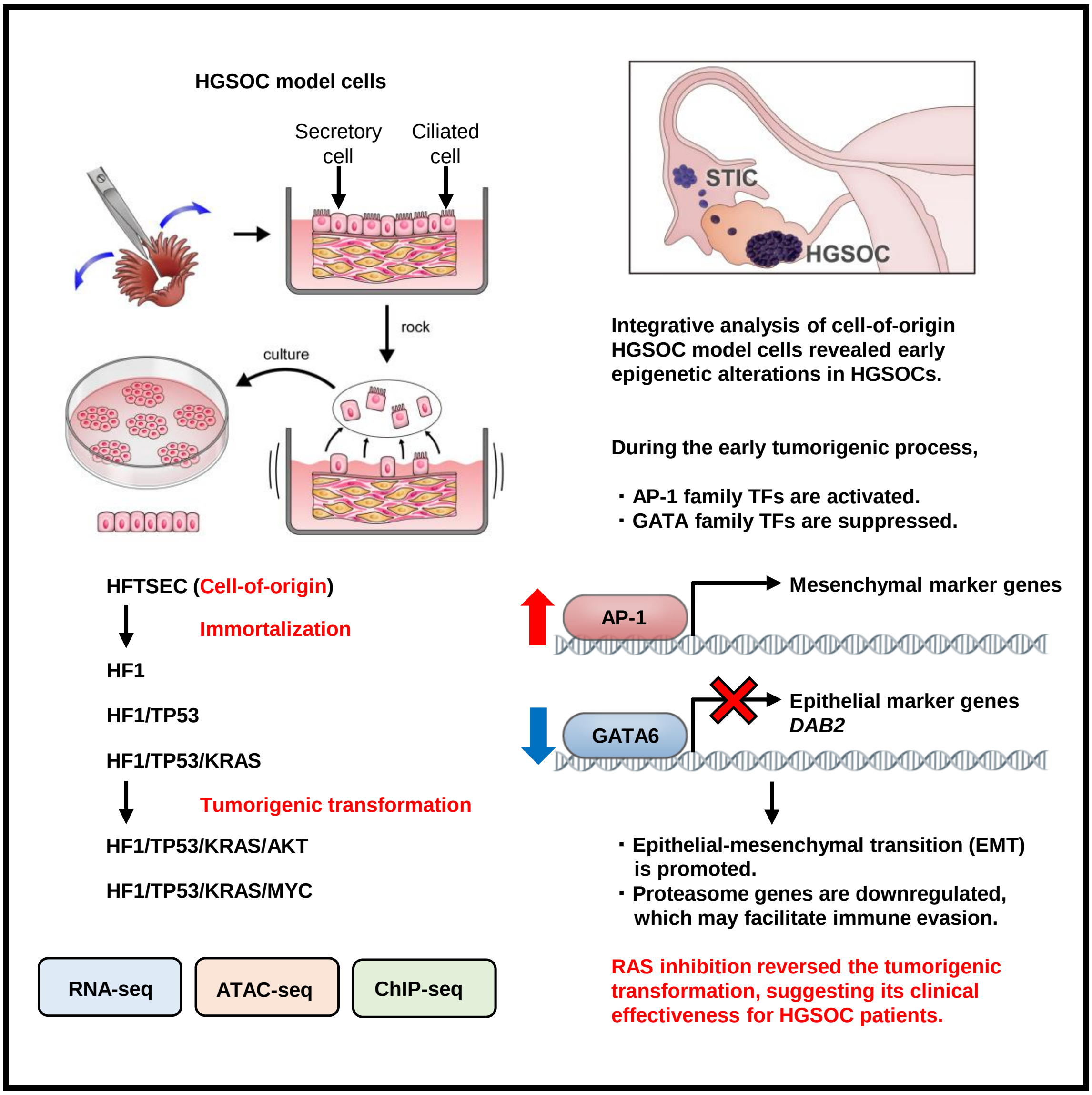

Supplementary Fig. 7 A graphical summary of the present study.
